# Treatment Patterns, Perceptions, Barriers, and Costs in Patients With Chronic Idiopathic Constipation in the United States

**DOI:** 10.1016/j.gastha.2026.100900

**Published:** 2026-02-17

**Authors:** Darren M. Brenner, Baharak Moshiree, Joanna de Courcy, Neil Reynolds, Teresa Taylor-Whiteley, Jeanne Jiang, Mei Lu, Brian Terreri, Eric D. Shah

**Affiliations:** 1 Feinberg School of Medicine, Northwestern University, Chicago, Illinois; 2 Atrium Health, Wake Forest University School of Medicine, Charlotte, North Carolina; 3 Adelphi Real World, Bollington, UK; 4 Takeda Pharmaceuticals USA, Inc, Lexington, Massachusetts; 5 Division of Gastroenterology and Hepatology, University of Michigan, Ann Arbor, Michigan

**Keywords:** Chronic Idiopathic Constipation, Treatment Patterns, Perceptions, Barriers, Costs

## Abstract

**Background and Aims:**

Limited real-world data are available on barriers to prescribing or recommending treatments for chronic idiopathic constipation (CIC), treatment experiences and expectations, and the financial impact of CIC from the perspectives of patients and health-care professionals (HCPs).

**Methods:**

In this noninterventional, cross-sectional, retrospective survey in the United States (October 2022–June 2023), board-certified gastroenterologists, motility specialists, advanced practice providers, and primary care physicians each recruited up to 8 adults with HCP-diagnosed CIC and no previous CIC clinical trial enrollment. HCP and patient surveys, and case report forms (CRFs) captured demographics and treatment patterns, perceptions, barriers, and costs.

**Results:**

Overall, 170 HCPs completed CRFs for 368 patients, of whom 230 completed the patient survey. Mean (standard deviation) patient age was 50.2 (16.5) years (CRF) and 49.7 (16.2) years (patient survey). HCPs would ideally recommend lifestyle/dietary modifications and over-the-counter treatments before prescription medications. Most HCPs rated increased quality of life (73.5%) and long-term efficacy (71.2%) as important when managing CIC, whereas most patients considered symptom relief (83.8%) and affordability (80.7%) as important. HCPs did not report substantial barriers to prescribing treatments, whereas patients reported difficulty getting an HCP appointment (36.3%) and a lack of awareness of CIC prescription medications (34.8%). Most HCPs and patients were satisfied with treatment options. The greatest patient expenses (30-day mean costs [$]) were seeing an HCP ($30.3) and prescription medication co-payments ($28.4).

**Conclusion:**

Despite most patients feeling satisfied with CIC treatment options, education on the availability of prescription medication in the United States is needed. This limited awareness, the patient expense of HCP visits, and prescription medication co-payments may limit patient access to CIC therapies.

## Introduction

Chronic idiopathic constipation (CIC), or functional constipation, is a common disorder of gut–brain interaction with no underlying physiological cause.^1^ Diagnosis is typically based on reports of difficult, infrequent, or incomplete defecation and/or hard stools, as classified by the Rome IV criteria.^2^^,^^3^ The prevalence of CIC in the United States is estimated to range from 9% to 20%.^4^ Prevalence is higher among women and patients with low socioeconomic status compared with men and patients with high socioeconomic status, respectively, and generally increases with age.^1^ Patients with CIC have reduced health-related quality of life and greater direct health-care costs than the general US population.^5^^,^^6^ Recent literature on the economic burden of CIC is limited; however, in 2010, average all-cause health-care costs for patients with CIC were estimated to be $9012 per person per year,^7^ and current costs are predicted to be higher.

An online US questionnaire completed by patients with CIC in 2017^8^ indicated that most patients managed their disease with lifestyle changes, such as increased fiber consumption and/or over-the-counter (OTC) laxatives, before consulting a health-care professional (HCP). This strategy aligns with the current clinical guidelines from the American Gastroenterological Association (2023) for the management of CIC^9^; however, data suggest only 40% of patients with CIC are satisfied with OTC treatments.^8^ In patients who do not respond or are intolerant to OTC treatments, the guidelines recommend the use of pharmacologic agents or prescription medications.^9^ Four prescription medications are now approved by the US Food and Drug Administration (FDA) specifically for the treatment of adults with CIC (linaclotide, lubiprostone, plecanatide, and prucalopride).^10^, ^11^, ^12^, ^13^ The efficacy and safety of these prescription medications in adult patients with CIC has been well established in double-blind, placebo-controlled, randomized clinical studies.^14^, ^15^, ^16^, ^17^ However, published data on the use of these therapies in real-world clinical practice are limited.

This study aimed to identify potential real-world barriers to prescribing or recommending treatments for CIC, to examine perceptions and expectations of these treatments, and to explore the financial impact of CIC on patients, from the perspectives of patients with CIC and their treating HCPs.

## Methods

### Study Design

This noninterventional, cross-sectional, retrospective survey was conducted in the United States between October 2022 and June 2023 and recruited adult patients with CIC and HCPs with patients with CIC in their care. The study design is presented in Supplementary Figure 1.

HCPs were eligible for enrollment in this study if they were board-certified gastroenterologists, motility specialists, advanced practice providers, or primary care physicians currently practicing in the United States, had made treatment decisions for more than 5 patients with CIC, had managed more than 10 patients with CIC in the past year, had more than 3 years’ experience in managing patients with CIC, and had access to patient medical records. Eligible HCPs were recruited via Survey Healthcare Globus and completed a 15-minute survey. This survey included questions on their demographics, treatment and prescribing patterns, perceptions and expectations of available CIC medications, and perceived treatment barriers.

Each HCP recruited up to 8 of the patients with whom they next consulted and who met the eligibility criteria. Patients were eligible for inclusion if they were aged at least 18 years, had an HCP diagnosis of CIC, could understand and provide informed consent, and were not previously nor currently enrolled in a clinical trial for CIC. Enrolled patients were asked to complete a 30-minute survey which included questions on their demographics, clinical status, experiences of the diagnostic and management journey, treatments, treatment barriers, perceptions of treatment attributes and effectiveness of CIC treatments, and patient expenses. For each patient, a case report form (CRF) was completed by their HCP (not all patients included in the CRF analysis completed the patient survey). The CRF included clinical information on patient demographics, clinical status, diagnostic journey, and treatment history. The risk of information and recall bias was mitigated by matching the patient survey to the CRF completed by the HCP.

### Study Outcomes

#### HCP and patient demographics

HCP demographics were captured in the HCP survey, and patient demographics were captured in the CRFs, both of which were completed by HCPs. A subset of the included patients also completed the patient survey; these patient demographics are reported separately.

#### Treatments for CIC

HCPs selected the lifestyle changes, OTC treatments, and prescription medications that they would be most likely to recommend in an ideal world as first-, second-, third-, and fourth-line treatments to patients with CIC from a list of 22 prespecified options (reported in Supplementary Table 1). HCPs also rated 22 CIC treatment attributes (including relief of symptoms, level of support and monitoring required, patient burden, cost, and safety) according to their importance in the treatment of their patients with CIC on a 7-point scale (Supplementary Table 2). Similarly, patients rated 6 attributes (affordability, ease of treatment administration, effectiveness in symptom relief, HCP recommendation, side effects, and short- and long-term safety) according to importance on a 7-point scale (Supplementary Table 3). In addition, HCPs recorded the first-, second-, third-, and fourth-line treatments being received by patients with CIC at the time of the study in the CRFs.

#### Treatment barriers

HCPs rated the most common barriers to prescribing CIC medications by allocating up to 100 points to each of the 13 attributes (0 = issue does not prevent prescribing treatments at all and 100 = issue completely prevents prescribing a treatment). Patients reported the most common barriers to receiving CIC treatments from a list of 10 prespecified scenarios (patient survey) and selected any that they had experienced in relation to their treatments for CIC.

#### HCP and patient perceptions of CIC prescription medication

HCPs and patients reported their perceptions of the effectiveness of 6 prespecified lifestyle or dietary modifications for relieving symptoms of CIC by ranking them using a 7-point scale (Supplementary Table 4). HCPs also reported their perceptions of 4 prescription medications for CIC: linaclotide, lubiprostone, plecanatide, and prucalopride. Nine attributes (overall satisfaction, access, cost, efficacy, HCP knowledge/understanding, insurance coverage, patient compliance, patient satisfaction, and safety) were rated on a 7-point scale (Supplementary Table 5). Patients also reported their overall satisfaction with current use of these same 4 prescription medications using a 7-point scale (Supplementary Table 6). Finally, patients reported their satisfaction with the treatments they were currently receiving or had previously taken for CIC. HCPs reported perceived patient satisfaction with the current or previously received CIC treatments in the CRF.

#### Financial impact of CIC

Patients reported direct and indirect expenses specifically related to CIC over the past 30 days for 7 prespecified potential sources of expenses. All costs are reported as US dollars.

A full list of survey questions presented to the HCPs and patients in this study are provided in Supplementary Tables 7 and 8, respectively.

### Data Analysis

Descriptive analyses (ie, mean [standard deviation, (SD)] or n [%]) were conducted using IBM Survey Reporter (version 7.5). No statistical testing was performed for the analyses reported. The risk of missing data was minimized by the inclusion of a progress bar in the online HCP survey so that HCPs could track their progress and completion of the survey. For the patient survey, short and concise instructions were provided, along with minimized logic, which helped to ensure that patients understood each question.

## Results

### HCP Demographics

In total, the survey was sent to 42,215 HCPs, of whom 361 were screened out and 170 completed responses for analysis. Most were primary care physicians/family practitioners (37.6% [64/170]) or general gastroenterologists (31.2% [53/170]). Most HCPs had more than 5 years’ experience in managing patients with CIC (86.5% [147/170]), and on average, HCPs had managed 214.5 patients with CIC in the past 12 months. Most HCPs were based in an urban practice (79.4% [135/170]) (Table 1).

**Table 1:** HCP Demographics and Characteristics

Demographic/characteristic	HCPs (N = 170)
Primary specialty, n (%)	
Primary care physician/family practitioner	64 (37.6)
General gastroenterologist	53 (31.2)
Nurse practitioner working in gastroenterology	16 (9.4)
Gastroenterologist motility specialist	12 (7.1)
Nurse practitioner working in primary care	12 (7.1)
Physician assistant working in primary care	7 (4.1)
Physician assistant working in gastroenterology	6 (3.5)
Number of patients with CIC managed over the past 12 mo, mean (SD)	214.5 (239.6)
Time spent managing patients with CIC, n (%)	
Fewer than 3 y	0 (0.0)
3–5 y	23 (13.5)
More than 5 y	147 (86.5)
Practice setting, n (%)	
Outpatient clinic/office/family medicine center	73 (42.9)
Community hospital	35 (20.6)
Academic center	18 (10.6)
Private center	12 (7.1)
Government or Veterans Affairs hospital	1 (0.6)
Long-term care facility or nursing home	1 (0.6)
Other	30 (17.6)
Practice location, n (%)	
Urban	135 (79.4)
Rural	35 (20.6)

Data were collected via the HCP survey.

### Patient Demographics

Overall, HCPs completed CRFs for 368 patients; of those, 230 patients completed the patient survey. The mean (SD) age of patients from the CRF and patients who completed the survey was 50.2 (16.5) years and 49.7 (16.2) years, respectively; most patients were female (CRF, 61.1% [225/368]; patient survey, 64.3% [148/230]). Most patients were White (CRF, 64.1% [236/368]; patient survey, 68.3% [157/230]) or Black or African American (CRF, 21.2% [78/368]; patient survey, 16.1% [37/230]). Overall, 13.6% (50/368) and 14.3% (33/230) of patients included in the CRF and those who completed the survey, respectively, were of Hispanic, Latin, or Spanish origin. Patients generally worked full time (CRF: 50.8% [187/368]; patient survey: 54.3% [125/230]) and had employer-provided/sponsored (52.3% [114/218]) or Medicare/Medicaid health insurance coverage (32.1% [70/218]) (patient survey). The demographics of the CRF patient population and those patients who completed the survey were generally similar (Table 2).

**Table 2:** Patient Demographics and Characteristics

Demographic/Characteristic	All Patients (CRF) (N = 368)	Survey Respondents (N = 230)^a^
Age, y, mean (SD)	50.2 (16.5)	49.7 (16.2)
Sex, n (%)		
Female	225 (61.1)	148 (64.3)
Male	141 (38.3)	82 (35.7)
Not reported	2 (0.5)	0 (0.0)
BMI, kg/m^2^, mean (SD)	26.9 (4.9)	–
Race/ethnicity, n (%)^b^		
White	236 (64.1)	157 (68.3)
Black or African American	78 (21.2)	37 (16.1)
South Asian (Indian subcontinent)	20 (5.4)	16 (7.0)
East or Southeast Asian	12 (3.3)	6 (2.6)
American Indian, Indigenous American, or Alaska Native	6 (1.6)	0 (0.0)
Middle Eastern or North African	5 (1.4)	3 (1.3)
Native Hawaiian or Pacific Islander	1 (0.3)	0 (0.0)
Other	15 (4.1)	22 (9.6)
Ethnicity, n (%)		
Hispanic, Latin, or Spanish origin	50 (13.6)	33 (14.3)
Employment/work status, n (%)		
Working full time	187 (50.8)	125 (54.3)
Retired	62 (16.8)	47 (20.4)
Working part time	53 (14.4)	26 (11.3)
Homemaker	24 (6.5)	15 (6.5)^c^
Unemployed	15 (4.1)	5 (2.2)^d^
Student	9 (2.4)	6 (2.6)^e^
On long-term sick leave	4 (1.1)	–
Not reported	14 (3.8)	6 (2.6)
Health insurance coverage, n (%)^f^		
Employer-provided/sponsored	–	114 (52.3)^g^
Medicare/Medicaid	–	70 (32.1)^h^
Privately arranged	–	23 (10.6)
Other	–	5 (2.3)^i^
None	–	6 (2.8)
No answer provided	–	12 (5.5)

Data were collected via the CRF or the patient survey.

BMI, body mass index.

a Not all patients included in the CRF analysis completed the patient survey.

b More than one response option could be selected.

c Full-time homemakers.

d Unemployed for reasons related to CIC.

e Full-time student, n = 5 (2.2%); part-time student, n = 1 (0.4%).

f N = 218.

g Via patient’s employer, n = 91 (41.7%); via partner/family’s employer, n = 23 (10.6%).

h Medicare, n = 28 (12.8%); Medicaid (or equivalent in state), n = 23 (10.6%); Medicare Advantage, n = 10 (4.6%); Medicare Part D prescription drug plan, n = 9 (4.1%).

i Health insurance exchange plan, n = 4 (1.8%); TRICARE/Veterans Health Care, n = 1 (0.5%).

### Treatments for CIC

**Figure 1:**
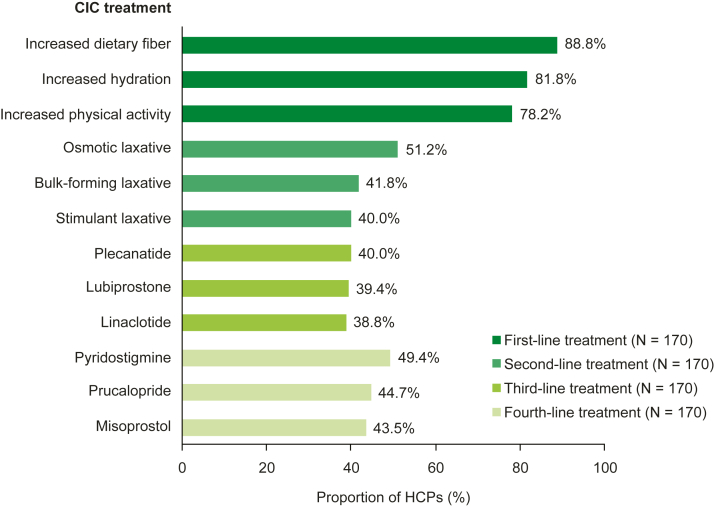
The 3 most commonly reported treatments that HCPs would recommend in an ideal world for patients with CIC for first-, second-, third-, and fourth-line treatments. Data were collected via the HCP survey. For each line of treatment, HCPs could select more than 1 treatment from 22 options: lifestyle or dietary modifications (increased dietary fiber, increased hydration, increased physical activity, introducing a schedule for using the toilet [habit training], using a toilet that is closer to the floor or adding a device to elevate the feet, using biofeedback methods); over-the-counter medications (bulk-forming laxatives, lactulose [generics], lubricant laxatives, osmotic laxatives, stimulant laxatives); prescription medications (colchicine [generics, Colcrys, Gloperba, Mitigare], dicyclomine hydrochloride [generics, Bentyl]^a^, hyoscyamine [Levsin], linaclotide [Linzess], lubiprostone [generic, Amitiza], misoprostol [generics, Cytotec], plecanatide [Trulance], prucalopride [Motegrity], pyridostigmine [Mestinon], tegaserod [Zelnorm]^a^, tenapanor [Ibsrela]^a^). The top 3 treatments for each treatment line are shown. ^a^Tegaserod and tenapanor are indicated for the treatment of irritable bowel syndrome with constipation in adult women aged <65 years and in adults, respectively, dicyclomine hydrochloride is indicated for the treatment of functional bowel/irritable bowel syndrome in adults, and colchicine is indicated for the treatment of gout flares and Familial Mediterranean Fever in adults and children aged ≥4 years; hence, these prescription medications were excluded from this table.^18^, ^19^, ^20^, ^21^

In an ideal world, HCPs were most likely to recommend increased dietary fiber (88.8% [151/170]), increased hydration (81.8% [139/170]), and increased physical activity (78.2% [133/170]) as first-line treatments and osmotic laxatives (51.2% [87/170]), bulk-forming laxatives (41.8% [71/170]), and stimulant laxatives (40.0% [68/170]) as second-line treatments (Figure 1). Overall, first- and second-line treatment recommendations were generally similar across HCP specialties, although general gastroenterologists and motility specialists were more likely to recommend prescription medications as a second-line treatment compared with other HCPs (Supplementary Table 1). Overall, HCPs were most likely to recommend prescription medications as third- and fourth-line treatments; however, this was generally driven by the prescribing habits of the advanced practice providers (Figure 1 and Supplementary Table 1).

Of the 368 patients included in the CRF, 303 (82.3%) patients were receiving a first-line treatment for CIC, most commonly bulk-forming laxatives (44.9% [136/303]), osmotic laxatives (44.2% [134/303]), and/or stimulant laxatives (36.3% [110/303]). Of these 303 patients, 201 (66.3%) were taking prescription medication as a first-line treatment, most commonly linaclotide (22.8% [69/303]) and lubiprostone (19.1% [58/303]). Overall, linaclotide and lubiprostone were also the most common first-line prescription medications provided across all HCP specialties, with the exception of motility specialists where prucalopride was the second most common prescription medication at first-line (Supplementary Table 9). In addition, HCPs reported that in patients who had never received the following prescription medications (linaclotide, lubiprostone, plecanatide, and prucalopride), a mean percentage of 37.0%–42.2% were clinically eligible (as determined by each HCP).

**Figure 2:**
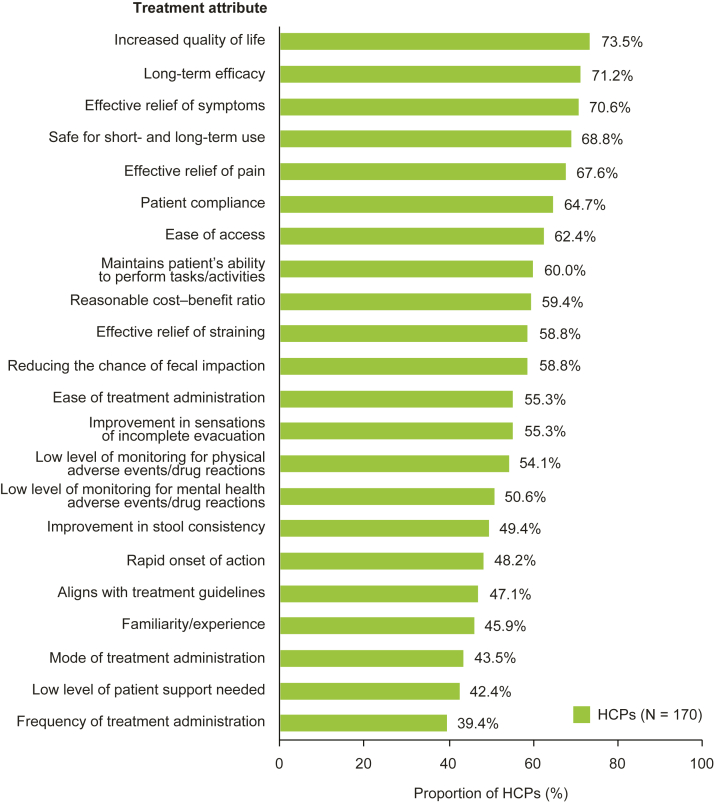
Proportions of HCPs who considered a CIC treatment attribute important when managing CIC. Data were collected via the HCP survey. For each option, HCPs could choose a rating from a 7-point scale: ‘not at all important,’ ‘low importance,’ ‘slightly important,’ ‘neutral,’ ‘moderately important,’ ‘very important,’ and ‘extremely important.’ Data are presented for attributes rated as ‘very important’ or ‘extremely important.’

When considering the management of patients with CIC, most HCPs rated increased quality of life (73.5% [125/170]), long-term efficacy (71.2% [121/170]), effective relief of symptoms (70.6% [120/170]), safe for short- and long-term use (68.8% [117/170]), effective relief of pain (67.6% [115/170]), patient compliance (64.7% [110/170]), and ease of access (62.4% [106/170]) as ‘very’ or ‘extremely’ important (Figure 2 and Supplementary Table 2). In the patient survey, symptom relief (83.8% [192/229]), affordability (80.7% [184/228]), and short- and long-term safety (77.3% [177/229]) were most frequently rated as ‘very’ or ‘extremely’ important when choosing a treatment for CIC (Supplementary Table 3).

### Treatment Barriers

Overall, HCPs did not report having substantial difficulties in prescribing treatments for CIC. HCPs found patient out-of-pocket costs (mean [SD] score out of 100: 18.8 [18.0]) and the complexity of getting insurance coverage (17.3 [16.2]) to be the greatest barriers; patient preference for OTC treatments (8.2 [8.9]) also made prescribing treatments difficult for HCPs. The barriers most commonly experienced by patients were difficulty getting an HCP appointment (36.3% [49/135]), lack of awareness that prescription medications were available for CIC (34.8% [47/135]), and unavailability of insurance for the CIC prescription medication (25.9% [35/135]) (Table 3).

**Table 3:** Experiences of Barriers to Treatment for CIC as Rated by HCPs and Patients

Barrier to Treatment	Experience
HCPs (N = 170)	Score out of 100, mean (SD)
Patient out-of-pocket cost for medications	18.8 (18.0)
Complexity of getting insurance coverage	17.3 (16.2)
Patient preference for over-the-counter options	8.2 (8.9)
Patient reluctant to take any medications at all for their condition	7.6 (10.7)
Concerns with efficacy	7.6 (9.8)
Concerns with safety	7.2 (9.9)
Patient did not want to take the medication prescribed to them	6.7 (7.5)
Lack of experience prescribing certain medications	6.2 (8.1)
Delays in diagnosis of CIC	5.3 (6.8)
Lack of evidence supporting their use	4.9 (7.4)
Negative experience using prescription medications for previous patients	4.9 (5.7)
Unaware of available treatment options	4.3 (6.8)
Other^a^	1.1 (3.0)


Patients (N = 135)	Proportion of Patients, n (%)
Difficulty getting an HCP appointment	49 (36.3)
Not aware that prescription medications were available for CIC	47 (34.8)
CIC prescription medication was not covered by insurance	35 (25.9)
Having to travel a long way to see an HCP	26 (19.3)
Out-of-pocket costs for the prescription medication too high	15 (11.1)
HCP appointment too expensive	12 (8.9)
Issues accessing online/telemedicine appointments and not being able to attend face-to-face appointments with an HCP due to COVID restrictions	12 (8.9)
Travel to see an HCP is expensive	8 (5.9)
HCP was reluctant to prescribe a medication	4 (3.0)
HCP did not know about the prescription treatment options	3 (2.2)

Data were collected via the HCP survey and the patient survey. The HCP survey collected data for each barrier using a 100-point scoring system: 0 = the issue does not prevent prescribing treatments at all; 100 = the issue completely prevents prescribing treatments. The patient survey collected data for 10 scenarios by patients selecting those that they had experienced.

COVID, coronavirus disease.

a There was no option in the survey for HCPs to provide further information.

### HCP and Patient Perceptions of CIC Treatments

**Figure 3:**
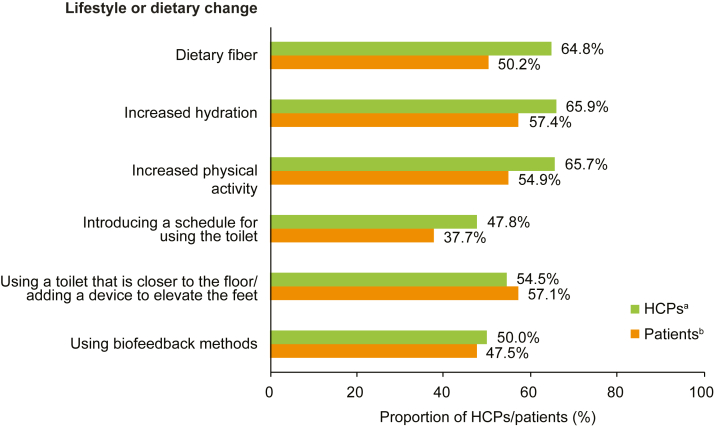
Proportions of patients and HCPs who considered a lifestyle or dietary modification effective in relieving symptoms of CIC. ^a^N = 170. ^b^Dietary fiber, N = 324; increased hydration, N = 314; increased physical activity, N = 280; introducing a schedule for using the toilet, N = 134; using a toilet that is closer to the floor/adding a device to elevate the feet, N = 99; using biofeedback methods, N = 60. Data were collected via the CRF and the patient survey. HCPs could choose a rating from a 7-point scale: ‘extremely ineffective,’ ‘very ineffective,’ ‘somewhat ineffective,’ ‘neutral,’ ‘somewhat effective,’ ‘very effective,’ and ‘extremely effective;’ data are presented for attributes rated as ‘somewhat effective,’ ‘very effective,’ and ‘extremely effective.’ Patients could choose a rating from a 7-point scale: ‘not at all effective,’ ‘low effectivity,’ ‘slightly effective,’ ‘neutral,’ ‘moderately effective,’ ‘very effective,’ and ‘extremely effective;’ data are presented for attributes rated as ‘moderately effective,’ ‘very effective,’ and ‘extremely effective.’

Most patients were using a lifestyle change to treat their CIC at the time of study. The most common included drinking more water (86.3% [189/219]), increasing dietary fiber (80.8% [177/219]), and exercise (63.9% [140/219]). Overall, higher proportions of HCPs than patients reported lifestyle changes as effective in relieving the symptoms of CIC (except for a toilet that is closer to the floor/elevation device) (Figure 3). Of the 6 lifestyle changes assessed for CIC, increased hydration was considered effective the most frequently by HCPs and patients (65.9% [207/314] of HCPs rated this as ‘somewhat’ to ‘extremely’ effective; 57.4% [124/216] of patients rated this as ‘moderately’ to ‘extremely’ effective). A toilet schedule was considered effective the least frequently (47.8% [64/134] of HCPs rated this as ‘somewhat’ to ‘extremely’ effective; 37.7% [29/77] of patients rated this as ‘moderately’ to ‘extremely’ effective) (Figure 3 and Supplementary Table 4).

**Figure 4:**
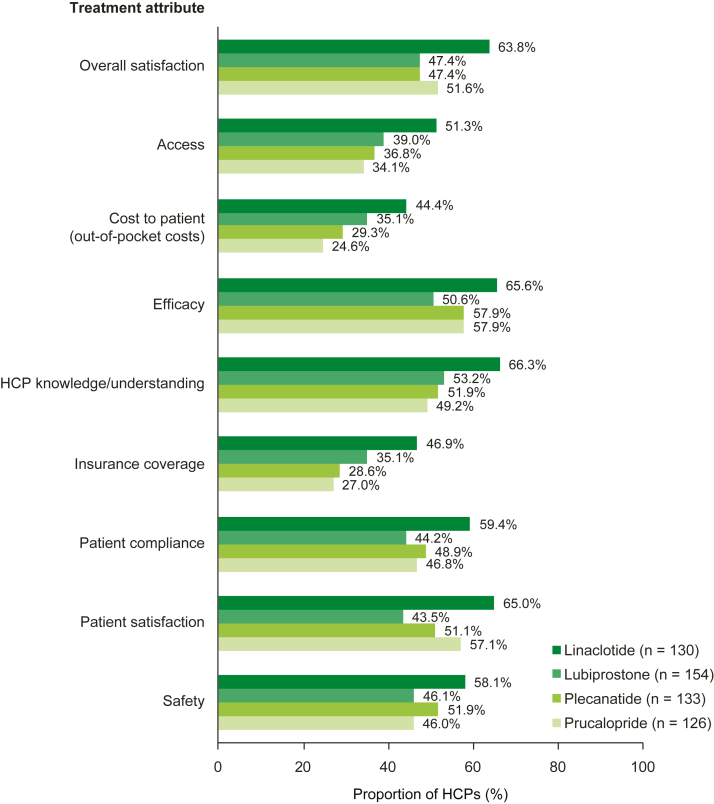
Proportions of HCPs who were positive about a CIC prescription medication, overall and by treatment attribute. Data were collected via the HCP survey. HCPs could choose ratings from a 7-point scale: ‘very negative,’ ‘negative,’ ‘somewhat negative,’ ‘neutral,’ ‘somewhat positive,’ ‘positive,’ and ‘very positive.’ Data are reported as the proportion of HCPs who rated the attribute as ‘positive’ or ‘very positive.’

Regarding the 4 prescription medications for CIC, a higher proportion of HCPs rated linaclotide (63.8% [102/160]) as ‘positive’ and ‘very positive’ in terms of their overall satisfaction in comparison with prucalopride (51.6% [65/126]), lubiprostone (47.4% [73/154]), and plecanatide (47.4% [63/133]). Findings were similar for the following attributes: access, cost to patient (out-of-pocket costs), efficacy, HCP knowledge/understanding, insurance coverage, patient compliance, patient satisfaction, and safety (Figure 4 and Supplementary Table 5). Most patients were satisfied with the 4 prescription medications examined, with a slightly higher proportion of patients ‘somewhat’ to ‘completely’ satisfied with lubiprostone (100.0% [25/25]) than with plecanatide (89.7% [26/29]), linaclotide (89.0% [65/73]), and prucalopride (85.7% [12/14]) (Supplementary Table 6).

### Financial Impact of CIC

In the 30 days before the study, the greatest patient expenses (mean [SD]) were related to seeing an HCP ($30.3 [$49.4]), co-payment for prescription medications ($28.4 [$40.0]), and OTC treatments ($24.3 [$20.3]) (Supplementary Table 10). Patients insured by Medicare experienced nearly double the cost of OTC treatments ($27.3 [$18.6], n = 26 vs $11.2 [$13.1], n = 20, respectively) and prescription medications ($20.9 [$43.0], n = 17 vs $11.8 [$17.9], n = 19), respectively) than those insured by Medicaid; similar trends were observed for Medicare Part D and Medicare Advantage. Patients with employer-provided insurance (n = 86) reported a 30-day cost for prescription medications of $34.7 ($48.7). The cost for seeing an HCP with Medicare and Medicaid were similar ($13.3 [$18.2], n = 18 vs $12.4 [$27.3], n = 18, respectively).

## Discussion

There are limited real-world data available on patients’ and HCPs’ experiences and perceptions of the CIC treatment landscape, the barriers to receiving treatment for CIC, and up-to-date associated health-care costs. When asked what they would recommend for patients with CIC in an ideal world, HCPs reported that they would initially recommend lifestyle or dietary modifications, then OTC treatments, which would subsequently be escalated to prescription medications. Overall, most patients and HCPs were satisfied with the treatment options available for CIC, although there were some distinct differences between patient and HCP perceptions of the importance of specific treatment attributes in disease management. Most HCPs rated increased quality of life and long-term efficacy as the important attributes in the management of patients with CIC, whereas patients considered symptom relief and treatment affordability as the most important attributes when choosing a treatment for CIC. Out-of-pocket costs and difficulty obtaining insurance coverage were reported as the most common barriers to prescribing CIC medications by HCPs, but patients reported difficulty getting an HCP appointment and lack of awareness of prescription medications for CIC.

The HCPs in this study were generally found to follow current treatment guidelines for CIC^9^ by initially recommending lifestyle modifications as first-line treatment options and then escalating to OTC treatments. Despite insufficient evidence in the literature,^9^^,^^22^ more than 80% of HCPs would recommend increased hydration as a first-line treatment for CIC. Overall, a higher proportion of HCPs than patients reported lifestyle modifications as effective in relieving symptoms of CIC, including consumption of dietary fiber and increased hydration. These data indicate discordance between HCP and patient perceptions of the benefits of lifestyle modifications and suggest that such treatments may not be effective for all patients. There is the possibility, however, of a selection bias in this consulting population because patients who find dietary and lifestyle modifications effective may be less likely to consult with their HCP.

In an ideal world, HCPs reported that they would escalate to prescription medications as third- and fourth-line interventions. However, the responses from the HCPs suggested that approximately 40% of the patients who had never received prescription medication for CIC were clinically eligible to do so (as determined by each HCP). Although this could be attributed to successful treatment with nonprescription therapies, this could also indicate wider accessibility issues. A lack of awareness of available prescription medications was identified as the most common barrier to treatment by patients, suggesting that further patient and HCP education would be beneficial. In addition, HCPs identified the complexity of obtaining insurance coverage as a common barrier to prescribing CIC medications, and a 2021 study found that the optimal cost-effective CIC treatment algorithm from the insurer’s perspective was to deny access to prescription drugs regardless of whether patients failed OTC treatments.^23^ The cost for co-payment for prescription medications was also identified in our study as one of the greatest expenses for patients with CIC. These data suggest that cost should be addressed at a policy level to ensure that patients have access to optimal treatments.

When comparing the 4 CIC prescription medications in terms of overall satisfaction, HCPs rated linaclotide slightly more positively than lubiprostone, plecanatide, and prucalopride. Linaclotide was approved by the US FDA for CIC in 2012,^10^ whereas plecanatide and prucalopride are newer drugs to the US market, having received FDA approval in 2017 and 2018, respectively.^12^^,^^13^ It is therefore possible that HCPs do not have the extent of knowledge of and experience with plecanatide and prucalopride that they do with linaclotide and thus may be less likely to prescribe these medications. These data could also suggest linaclotide is more easily accessible than the newly approved medications, and access could be restricted by step therapies. HCPs perceived linaclotide more positively in terms of safety than the 3 prescription comparators. One factor that may potentially be contributing to this is the multiple dosing strengths available to HCPs when prescribing linaclotide.^10^ Statistical testing was not performed for these analyses, and therefore, any comparisons made between the HCPs’ perceptions of the 4 prescription medications are descriptive. Notably, lubiprostone, plecanatide, and prucalopride are not currently FDA-approved for patients aged <18 years with CIC.^11^, ^12^, ^13^ Therefore, assessing these therapeutics in a pediatric population is not currently possible. Real-world data on the pediatric CIC treatment landscape, from the perspectives of patients and their HCPs, are needed.

Overall, more than 80% of patients were somewhat to completely satisfied with the prescription medications available for CIC; this is considerably higher than previously reported in the literature.^8^^,^^24^ A US patient questionnaire conducted in 2016 reported less than half (41%) of patients with CIC were satisfied or completely satisfied with their branded prescription medication owing to lack of efficacy and side effects.^8^ This 2016 study was conducted before the US FDA approval of plecanatide and prucalopride^12^^,^^13^; thus, the notable increase in patient satisfaction observed in our study could therefore be attributed to the availability and efficacy of additional prescription medication options for CIC.

This study has multiple strengths, including the presentation of real-world US treatment data from a large patient population with insights from a diverse HCP population, including from multiple specialties. Another strength of this study is that patients were enrolled prospectively as the next eligible patients with whom the HCP consulted, which ensured a heterogeneous patient population and generalizability of the data. Limited patient inclusion and exclusion criteria also allowed an assessment of a wide spectrum of CIC, including patients with milder symptoms.

The limitations of this study include potential selection bias which arose from some data only being available from consulting patients who chose to complete the survey. These individuals may be more engaged than the general CIC population. No statistical testing was performed, and therefore, the analyses were descriptive. In addition, patients completed a paper survey, which presents a higher likelihood of missed questions than online surveys.^25^ Recall and information bias can exist with self-reported research,^26^ but matching of some of the CRF data with the patient survey responses helped to mitigate these risks. The surveys and CRF were designed specifically for this study and have not been validated for future/repeated use. Some patients included in the CRF analysis did not return the completed patient survey. Finally, standardized data collection techniques and consistent, neutral questioning helped to further mitigate the risk of information bias.

## Conclusion

HCPs were generally found to follow current CIC clinical treatment guidelines by escalating CIC management from lifestyle/dietary modifications and OTC treatments to prescription medications. Most patients were satisfied with CIC treatment options, particularly with prescription medications. HCPs were generally more satisfied than patients with the use of lifestyle modifications in the treatment of CIC. Approximately 40% of the patients who had never received prescription medications for CIC were clinically eligible to do so, which may indicate wider accessibility issues. Patients also identified a lack of awareness of prescription medications and limited insurance coverage as common barriers to receiving these medications, whereas HCPs identified patient out-of-pocket costs and the complexity of getting insurance coverage as common barriers to prescribing medications. These findings highlight the importance of patient and HCP education and cost discussions at the policy level to improve access to prescription therapies for patients with CIC.

## Supplementary Material

Supplementary Material

Extended PDF
