# Supplementary material for: Treatment Patterns, Perceptions, Barriers, and Costs in Patients With Chronic Idiopathic Constipation in the United States
Source: Gastro Hep Adv. 2026 Feb 17;5(4):100900. doi: 10.1016/j.gastha.2026.100900 (PMC13018922; doi:10.1016/j.gastha.2026.100900)
Supplement: Supplementary Material [file mmc1.pdf]

Supplementary Material

Treatment Patterns, Perceptions, Barriers, and Costs in Patients with Chronic Idiopathic Constipation in the United States

Darren M. Brenner,[1,*] Baharak Moshiree,[2,*] Joanna de Courcy,[3] Neil Reynolds,[3] Teresa Taylor-Whiteley,[3] Jeanne Jiang,[4] Mei Lu,[4] Brian Terreri,[4] and Eric D. Shah[5]

[1]Feinberg School of Medicine, Northwestern University, Chicago, Illinois; [2]Atrium Health, Wake Forest University School of Medicine, Charlotte, North Carolina; [3]Adelphi Real World, Bollington, UK; [4]Takeda Pharmaceuticals USA, Inc., Lexington, Massachusetts; and [5]Division of Gastroenterology and Hepatology, University of Michigan, Ann Arbor, Michigan

*Denotes co-first authorship.

Supplementary Figure 1. Study design.

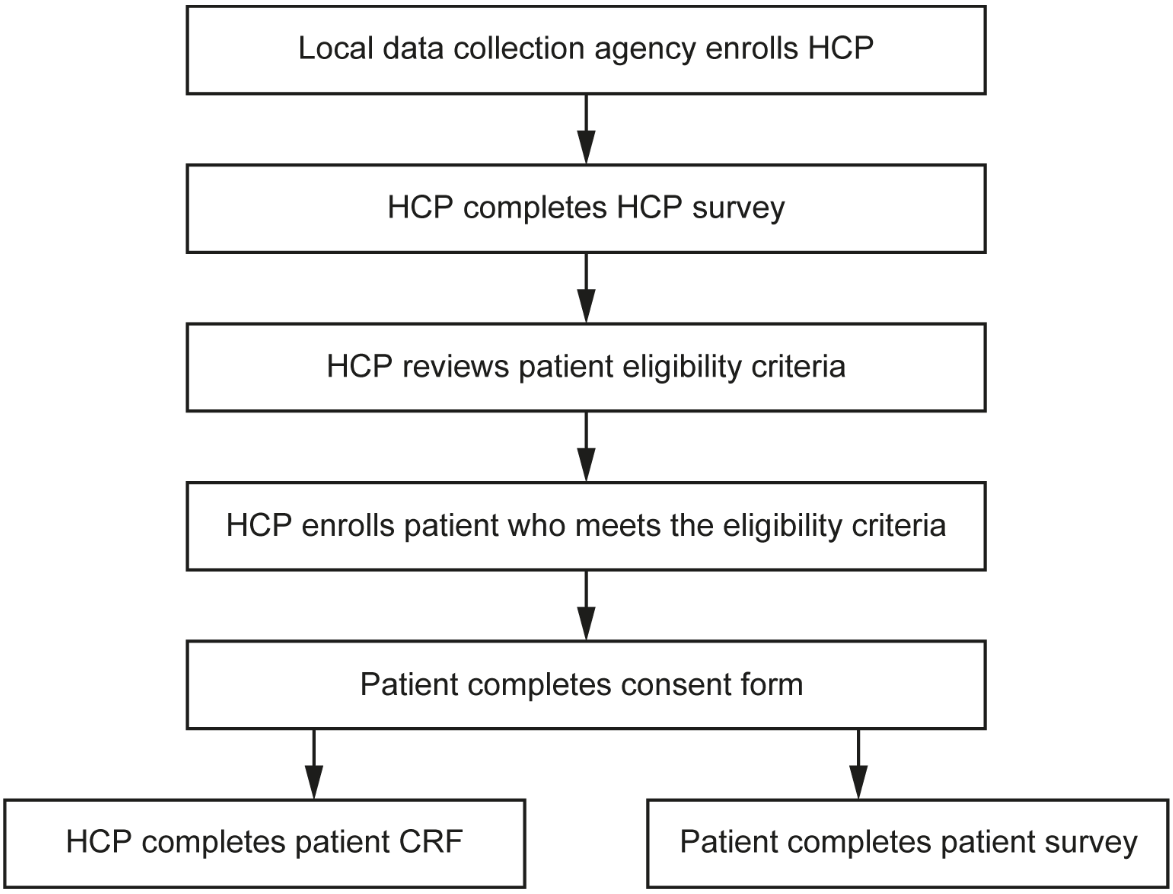

CRF, case report form; HCP, health-care professional.

Supplementary Table 1. Recommendations of CIC treatments by HCPs at first, second, third, and fourth lines in an ideal world, stratified by HCP specialty

Treatment, n (%)[a]	Total (N = 170)	General gastroenterologist (n = 53)	Motility specialist (n = 12)	Primary care physician (n = 64)	Advanced practice provider (n = 41)
**First-line treatment**					
Increased dietary fiber	151 (88.8)	43 (81.1)	12 (100.0)	58 (90.6)	38 (92.7)
Increased hydration	139 (81.8)	36 (67.9)	10 (83.3)	55 (85.9)	38 (92.7)
Increased physical activity	133 (78.2)	37 (69.8)	9 (75.0)	51 (79.7)	36 (87.8)
Introducing a schedule for using the toilet	98 (57.6)	31 (58.5)	7 (58.3)	32 (50.0)	28 (68.3)
Using a toilet that is closer to the floor/adding a device to elevate the feet	86 (50.6)	24 (45.3)	9 (75.0)	27 (42.2)	26 (63.4)
Bulk-forming laxatives	82 (48.2)	29 (54.7)	7 (58.3)	27 (42.2)	19 (46.3)
Using biofeedback methods	63 (37.1)	16 (30.2)	7 (58.3)	21 (32.8)	19 (46.3)
Osmotic laxatives	49 (28.8)	23 (43.4)	5 (41.7)	14 (21.9)	7 (17.1)
Stimulant laxatives	27 (15.9)	11 (20.8)	3 (25.0)	7 (10.9)	6 (14.6)
Lubricant laxatives	25 (14.7)	12 (22.6)	3 (25.0)	6 (9.4)	4 (9.8)
Lactulose	22 (12.9)	10 (18.9)	3 (25.0)	4 (6.3)	5 (12.2)
Hyoscyamine	14 (8.2)	8 (15.1)	1 (8.3)	5 (7.8)	0 (0.0)
Linaclotide	11 (6.5)	5 (9.4)	2 (16.7)	2 (3.1)	2 (4.9)
Plecanatide	9 (5.3)	4 (7.5)	1 (8.3)	3 (4.7)	1 (2.4)
Lubiprostone	7 (4.1)	5 (9.4)	0 (0.0)	1 (1.6)	1 (2.4)
Misoprostol	6 (3.5)	5 (9.4)	0 (0.0)	0 (0.0)	1 (2.4)
Prucalopride	6 (3.5)	4 (7.5)	1 (8.3)	1 (1.6)	0 (0.0)
Pyridostigmine	2 (1.2)	2 (3.8)	0 (0.0)	0 (0.0)	0 (0.0)
**Second-line treatment**					
Osmotic laxatives	87 (51.2)	20 (37.7)	6 (50.0)	33 (51.6)	28 (68.3)
Bulk-forming laxatives	71 (41.8)	21 (39.6)	6 (50.0)	22 (34.4)	22 (53.7)
Stimulant laxatives	68 (40.0)	22 (41.5)	5 (41.7)	21 (32.8)	20 (48.8)

Treatment, n (%)[a]	Total (N = 170)	General gastroenterologist (n = 53)	Motility specialist (n = 12)	Primary care physician (n = 64)	Advanced practice provider (n = 41)
Lubricant laxatives	63 (37.1)	16 (30.2)	4 (33.3)	22 (34.4)	21 (51.2)
Lactulose	49 (28.8)	16 (30.2)	7 (58.3)	15 (23.4)	11 (26.8)
Using biofeedback methods	48 (28.2)	12 (22.6)	6 (50.0)	15 (23.4)	15 (36.6)
Linaclotide	39 (22.9)	19 (35.8)	4 (33.3)	12 (18.8)	4 (9.8)
Lubiprostone	37 (21.8)	19 (35.8)	6 (50.0)	11 (17.2)	1 (2.4)
Using a toilet that is closer to the floor/adding a device to elevate the feet	36 (21.2)	12 (22.6)	4 (33.3)	9 (14.1)	11 (26.8)
Increased physical activity	31 (18.2)	14 (26.4)	6 (50.0)	5 (7.8)	6 (14.6)
Hyoscyamine	31 (18.2)	14 (26.4)	5 (41.7)	4 (6.3)	8 (19.5)
Plecanatide	29 (17.1)	14 (26.4)	3 (25.0)	7 (10.9)	5 (12.2)
Increased hydration	27 (15.9)	12 (22.6)	4 (33.3)	8 (12.5)	3 (7.3)
Introducing a schedule for using the toilet	27 (15.9)	11 (20.8)	5 (41.7)	6 (9.4)	5 (12.2)
Increased dietary fiber	25 (14.7)	11 (20.8)	4 (33.3)	6 (9.4)	4 (9.8)
Prucalopride	24 (14.1)	13 (24.5)	4 (33.3)	3 (4.7)	4 (9.8)
Pyridostigmine	12 (7.1)	5 (9.4)	2 (16.7)	3 (4.7)	2 (4.9)
Misoprostol	11 (6.5)	3 (5.7)	4 (33.3)	3 (4.7)	1 (2.4
**Third-line treatment**					
Plecanatide	68 (40.0)	23 (43.4)	4 (33.3)	22 (34.4)	19 (46.3)
Lubiprostone	67 (39.4)	14 (26.4)	4 (33.3)	23 (35.9)	26 (63.4)
Linaclotide	66 (38.8)	16 (30.2)	3 (25.0)	25 (39.1)	22 (53.7)
Lactulose	57 (33.5)	17 (32.1)	3 (25.0)	18 (28.1)	19 (46.3)
Prucalopride	56 (32.9)	19 (35.8)	3 (25.0)	14 (21.9)	20 (48.8)
Hyoscyamine	53 (31.2)	14 (26.4)	5 (41.7)	18 (28.1)	16 (39.0)
Stimulant laxatives	44 (25.9)	10 (18.9)	5 (41.7)	14 (21.9)	15 (36.6)
Misoprostol	43 (25.3)	13 (24.5)	4 (33.3)	12 (18.8)	14 (34.1)
Lubricant laxatives	42 (24.7)	13 (24.5)	5 (41.7)	9 (14.1)	15 (36.6)
Pyridostigmine	36 (21.2)	14 (26.4)	4 (33.3)	8 (12.5)	10 (24.4)

Treatment, n (%)[a]	Total (N = 170)	General gastroenterologist (n = 53)	Motility specialist (n = 12)	Primary care physician (n = 64)	Advanced practice provider (n = 41)
Osmotic laxatives	27 (15.9)	7 (13.2)	4 (33.3)	7 (10.9)	9 (22.0)
Using biofeedback methods	27 (15.9)	12 (22.6)	3 (25.0)	5 (7.8)	7 (17.1)
Increased hydration	25 (14.7)	11 (20.8)	6 (50.0)	6 (9.4)	2 (4.9)
Increased physical activity	19 (11.2)	10 (18.9)	3 (25.0)	4 (6.3)	2 (4.9)
Increased dietary fiber	16 (9.4)	5 (9.4)	3 (25.0)	5 (7.8)	3 (7.3)
Using a toilet that is closer to the floor/adding a device to elevate the feet	16 (9.4)	7 (13.2)	2 (16.7)	5 (7.8)	2 (4.9)
Bulk-forming laxatives	17 (10.0)	6 (11.3)	4 (33.3)	4 (6.3)	3 (7.3)
Introducing a schedule for using the toilet	14 (8.2)	4 (7.5)	3 (25.0)	5 (7.8)	2 (4.9)
**Fourth-line treatment**					
Pyridostigmine	84 (49.4)	19 (35.8)	5 (41.7)	26 (40.6)	34 (82.9)
Prucalopride	76 (44.7)	19 (35.8)	5 (41.7)	30 (46.9)	22 (53.7)
Misoprostol	74 (43.5)	19 (35.8)	5 (41.7)	21 (32.8)	29 (70.7)
Plecanatide	58 (34.1)	13 (24.5)	5 (41.7)	18 (28.1)	22 (53.7)
Lubiprostone	57 (33.5)	15 (28.3)	6 (50.0)	15 (23.4)	21 (51.2)
Linaclotide	55 (32.4)	16 (30.2)	4 (33.3)	17 (26.6)	18 (43.9)
Hyoscyamine	53 (31.2)	13 (24.5)	6 (50.0)	13 (20.3)	21 (51.2)
Lactulose	34 (20.0)	4 (7.5)	5 (41.7)	13 (20.3)	12 (29.3)
Using biofeedback methods	20 (11.8)	11 (20.8)	3 (25.0)	2 (3.1)	4 (9.8)
Lubricant laxatives	19 (11.2)	9 (17.0)	1 (8.3)	6 (9.4)	3 (7.3)
Stimulant laxatives	18 (10.6)	6 (11.3)	2 (16.7)	6 (9.4)	4 (9.8)
Introducing a schedule for using the toilet	16 (9.4)	6 (11.3)	5 (41.7)	3 (4.7)	2 (4.9)
Increased dietary fiber	15 (8.8)	7 (13.2)	3 (25.0)	4 (6.3)	1 (2.4)
Using a toilet that is closer to the floor/adding a device to elevate the feet	15 (8.8)	8 (15.1)	1 (8.3)	5 (7.8)	1 (2.4)
Increased hydration	14 (8.2)	6 (11.3)	3 (25.0)	4 (6.3)	1 (2.4)
Increased physical activity	12 (7.1)	6 (11.3)	3 (25.0)	2 (3.1)	1 (2.4)
Osmotic laxatives	11 (6.5)	5 (9.4)	3 (25.0)	2 (3.1)	1 (2.4)

Treatment, n (%)[a]	Total (N = 170)	General gastroenterologist (n = 53)	Motility specialist (n = 12)	Primary care physician (n = 64)	Advanced practice provider (n = 41)
Bulk-forming laxatives	9 (5.3)	3 (5.7)	1 (8.3)	4 (6.3)	1 (2.4)

Data were collected via the HCP survey. For each line of treatment, HCPs could select more than one treatment. Data are ordered by total frequency.

[a]Tegaserod and tenapanor are indicated for the treatment of irritable bowel syndrome with constipation in adult women aged < 65 years and adults, respectively, dicyclomine hydrochloride is indicated for the treatment of functional bowel/irritable bowel syndrome in adults, and colchicine is indicated for the treatment of gout flares and Familial Mediterranean Fever in adults and children aged ≥ 4 years; hence, these prescription medications were excluded from this table.[1-4]

CIC, chronic idiopathic constipation; HCP, health-care professional.

Supplementary Table 2. Rating of importance of attributes of CIC treatments by HCPs

Treatment attribute, n (%)	HCPs (N = 170)						
	Not at all important	Low importance	Slightly important	Neutral	Moderately important	Very important	Extremely important
Aligns with treatment guidelines	0 (0.0)	3 (1.8)	5 (2.9)	21 (12.4)	61 (35.9)	60 (35.3)	20 (11.8)
Ease of access	0 (0.0)	0 (0.0)	6 (3.5)	11 (6.5)	47 (27.6)	62 (36.5)	44 (25.9)
Ease of treatment administration	0 (0.0)	1 (0.6)	2 (1.2)	17 (10.0)	56 (32.9)	67 (39.4)	27 (15.9)
Effective relief of pain	0 (0.0)	0 (0.0)	5 (2.9)	13 (7.6)	37 (21.8)	75 (44.1)	40 (23.5)
Effective relief of straining	0 (0.0)	0 (0.0)	2 (1.2)	13 (7.6)	55 (32.4)	66 (38.8)	34 (20.0)
Effective relief of symptoms	0 (0.0)	0 (0.0)	1 (0.6)	13 (7.6)	36 (21.2)	74 (43.5)	46 (27.1)
Familiarity/experience	0 (0.0)	3 (1.8)	11 (6.5)	28 (16.5)	50 (29.4)	58 (34.1)	20 (11.8)
Frequency of treatment administration	0 (0.0)	6 (3.5)	13 (7.6)	18 (10.6)	66 (38.8)	45 (26.5)	22 (12.9)
Improvement in sensations of incomplete evacuation	0 (0.0)	1 (0.6)	5 (2.9)	15 (8.8)	55 (32.4)	64 (37.6)	30 (17.6)
Improvement in stool consistency	0 (0.0)	2 (1.2)	9 (5.3)	11 (6.5)	64 (37.6)	56 (32.9)	28 (16.5)
Increased quality of life	0 (0.0)	0 (0.0)	2 (1.2)	15 (8.8)	28 (16.5)	59 (34.7)	66 (38.8)
Long-term efficacy	0 (0.0)	0 (0.0)	4 (2.4)	9 (5.3)	36 (21.2)	77 (45.3)	44 (25.9)
Low level of monitoring for mental health adverse events/drug reactions	1 (0.6)	2 (1.2)	7 (4.1)	31 (18.2)	43 (25.3)	63 (37.1)	23 (13.5)
Low level of monitoring for physical adverse events/drug reactions	0 (0.0)	3 (1.8)	9 (5.3)	16 (9.4)	50 (29.4)	70 (41.2)	22 (12.9)
Low level of patient support needed	0 (0.0)	0 (0.0)	8 (4.7)	34 (20.0)	56 (32.9)	52 (30.6)	20 (11.8)
Maintains patient's ability to perform tasks/activities	0 (0.0)	0 (0.0)	8 (4.7)	17 (10.0)	43 (25.3)	65 (38.2)	37 (21.8)

Treatment attribute, n (%)	HCPs (N = 170)						
	**Not at all important**	**Low importance**	**Slightly important**	**Neutral**	**Moderately important**	**Very important**	**Extremely important**
Mode of treatment administration	0 (0.0)	1 (0.6)	12 (7.1)	23 (13.5)	60 (35.3)	55 (32.4)	19 (11.2)
Patient compliance	0 (0.0)	0 (0.0)	3 (1.8)	19 (11.2)	38 (22.4)	72 (42.4)	38 (22.4)
Rapid onset of action	1 (0.6)	2 (1.2)	10 (5.9)	21 (12.4)	54 (31.8)	59 (34.7)	23 (13.5)
Reasonable cost–benefit ratio	1 (0.6)	0 (0.0)	7 (4.1)	14 (8.2)	47 (27.6)	65 (38.2)	36 (21.2)
Reducing the chance of fecal impaction	0 (0.0)	1 (0.6)	7 (4.1)	13 (7.6)	49 (28.8)	62 (36.5)	38 (22.4)
Safe for short- and long-term use	0 (0.0)	1 (0.6)	5 (2.9)	13 (7.6)	34 (20.0)	63 (37.1)	54 (31.8)

Data were collected via the HCP survey.

CIC, chronic idiopathic constipation; HCP, health-care professional.

Supplementary Table 3. Rating of importance of attributes of CIC treatments by patients

Treatment attribute, n (%)	Patients (N = 230)						
	Extremely unimportant	Very unimportant	Somewhat unimportant	Neutral	Somewhat important	Very important	Extremely important
Affordable treatment (n = 228)	1 (0.4)	1 (0.4)	2 (0.9)	13 (5.7)	27 (11.8)	64 (28.1)	120 (52.6)
Ease of treatment administration (n = 227)	0 (0.0)	4 (1.8)	7 (3.1)	13 (5.7)	28 (12.3)	71 (31.3)	104 (45.8)
Effective relief of symptoms (n = 229)	0 (0.0)	1 (0.4)	1 (0.4)	16 (7.0)	19 (8.3)	78 (34.1)	114 (49.8)
HCP recommendation based on patient's previous experience (n = 227)	0 (0.0)	2 (0.9)	9 (4.0)	32 (14.1)	36 (15.9)	59 (26.0)	89 (39.2)
Low number of side effects (n = 229)	0 (0.0)	3 (1.3)	1 (0.4)	16 (7.0)	32 (14.0)	63 (27.5)	114 (49.8)
Safe for short- and long-term use (n = 229)	0 (0.0)	1 (0.4)	6 (2.6)	11 (4.8)	34 (14.8)	59 (25.8)	118 (51.5)

Data were collected via the patient survey.

CIC, chronic idiopathic constipation.

Supplementary Table 4. Rating of effectiveness of lifestyle or dietary modifications in relieving symptoms of CIC by HCPs and patients

Lifestyle/dietary modification, n (%)	CRF (N = 368)						
	Extremely ineffective	Very ineffective	Somewhat ineffective	Neutral	Somewhat effective	Very effective	Extremely effective
Dietary fiber (n = 324)	6 (1.9)	20 (6.2)	47 (14.5)	38 (11.7)	112 (34.6)	72 (22.2)	26 (8.0)
Increased hydration (n = 314)	3 (1.0)	12 (3.8)	43 (13.7)	46 (14.6)	103 (32.8)	68 (21.7)	36 (11.5)
Increased physical activity (n = 280)	2 (0.7)	14 (5.0)	33 (11.8)	33 (11.8)	103 (36.8)	55 (19.6)	26 (9.3)
Introducing schedule for using the toilet (n = 134)	3 (2.2)	10 (7.5)	19 (14.2)	33 (24.6)	43 (32.1)	16 (11.9)	5 (3.7)
Using a toilet that is closer to the floor/adding a device to elevate the feet (n = 99)	1 (1.0)	1 (1.0)	10 (10.1)	27 (27.3)	35 (35.4)	15 (15.2)	4 (4.0)
Using biofeedback methods (n = 60)	1 (1.7)	7 (11.7)	8 (13.3)	9 (15.0)	17 (28.3)	9 (15.0)	4 (6.7)
	**Patients (N = 230)**						
	**Not at all effective**	**Low effectivity**	**Slightly effective**	**Neutral**	**Moderately effective**	**Very effective**	**Extremely effective**
Dietary fiber (n = 215)	8 (3.7)	26 (12.1)	41 (19.1)	32 (14.9)	65 (30.2)	29 (13.5)	14 (6.5)
Increased hydration (n = 216)	6 (2.8)	17 (7.9)	37 (17.1)	32 (14.8)	59 (27.3)	37 (17.1)	28 (13.0)
Increased physical activity (n = 184)	7 (3.8)	19 (10.3)	28 (15.2)	29 (15.8)	53 (28.8)	29 (15.8)	19 (10.3)
Introducing schedule for using the toilet (n = 77)	7 (9.1)	9 (11.7)	12 (15.6)	20 (26.0)	25 (32.5)	2 (2.6)	2 (2.6)
Using a toilet that is closer to the floor/adding a device to elevate the feet (n = 70)	2 (2.9)	4 (5.7)	11 (15.7)	13 (18.6)	25 (35.7)	12 (17.1)	3 (4.3)
Using biofeedback methods (n = 40)	5 (12.5)	3 (7.5)	5 (12.5)	8 (20.0)	9 (22.5)	8 (20.0)	2 (5.0)

Data were collected via the CRF and the patient survey.

CIC, chronic idiopathic constipation; CRF, case report form; HCP, health-care professional.

Supplementary Table 5. Perceptions of four CIC prescription medications: linaclotide, lubiprostone, plecanatide, and prucalopride by HCPs

Treatment attribute, n (%)[a]	Very negative	Negative	Somewhat negative	Neutral	Somewhat positive	Positive	Very positive
**Overall satisfaction**							
Linaclotide	0 (0.0)	1 (0.6)	3 (1.9)	17 (10.6)	37 (23.1)	72 (45.0)	30 (18.8)
Lubiprostone	0 (0.0)	0 (0.0)	11 (7.1)	24 (15.6)	46 (29.9)	57 (37.0)	16 (10.4)
Plecanatide	0 (0.0)	0 (0.0)	4 (3.0)	14 (10.5)	52 (39.1)	45 (33.8)	18 (13.5)
Prucalopride	1 (0.8)	0 (0.0)	8 (6.3)	20 (15.9)	32 (25.4)	50 (39.7)	15 (11.9)
**Access**							
Linaclotide	1 (0.6)	6 (3.8)	7 (4.4)	23 (14.4)	41 (25.6)	59 (36.9)	23 (14.4)
Lubiprostone	1 (0.6)	7 (4.5)	13 (8.4)	29 (18.8)	44 (28.6)	42 (27.3)	18 (11.7)
Plecanatide	4 (3.0)	5 (3.8)	18 (13.5)	19 (14.3)	38 (28.6)	33 (24.8)	16 (12.0)
Prucalopride	3 (2.4)	6 (4.8)	21 (16.7)	16 (12.7)	37 (29.4)	32 (25.4)	11 (8.7)
**Cost to patient (out-of-pocket costs)**							
Linaclotide	1 (0.6)	7 (4.4)	15 (9.4)	26 (16.3)	40 (25.0)	55 (34.4)	16 (10.0)
Lubiprostone	2 (1.3)	8 (5.2)	19 (12.3)	32 (20.8)	39 (25.3)	36 (23.4)	18 (11.7)
Plecanatide	5 (3.8)	7 (5.3)	23 (17.3)	18 (13.5)	41 (30.8)	26 (19.5)	13 (9.8)
Prucalopride	5 (4.0)	8 (6.3)	24 (19.0)	24 (19.0)	34 (27.0)	22 (17.5)	9 (7.1)
**Efficacy**							
Linaclotide	0 (0.0)	0 (0.0)	0 (0.0)	13 (8.1)	42 (26.3)	74 (46.3)	31 (19.4)
Lubiprostone	0 (0.0)	0 (0.0)	6 (3.9)	14 (9.1)	56 (36.4)	62 (40.3)	16 (10.4)
Plecanatide	0 (0.0)	0 (0.0)	3 (2.3)	15 (11.3)	38 (28.6)	59 (44.4)	18 (13.5)
Prucalopride	1 (0.8)	0 (0.0)	6 (4.8)	14 (11.1)	32 (25.4)	57 (45.2)	16 (12.7)
**HCP knowledge/understanding**							
Linaclotide	0 (0.0)	1 (0.6)	5 (3.1)	13 (8.1)	35 (21.9)	64 (40.0)	42 (26.3)
Lubiprostone	0 (0.0)	0 (0.0)	5 (3.2)	21 (13.6)	46 (29.9)	49 (31.8)	33 (21.4)
Plecanatide	0 (0.0)	0 (0.0)	6 (4.5)	20 (15.0)	38 (28.6)	44 (33.1)	25 (18.8)
Prucalopride	1 (0.8)	0 (0.0)	5 (4.0)	21 (16.7)	37 (29.4)	39 (31.0)	23 (18.3)
**Insurance coverage**							
Linaclotide	2 (1.3)	6 (3.8)	12 (7.5)	19 (11.9)	46 (28.8)	54 (33.8)	21 (13.1)
Lubiprostone	3 (1.9)	6 (3.9)	17 (11.0)	28 (18.2)	46 (29.9)	36 (23.4)	18 (11.7)
Plecanatide	5 (3.8)	8 (6.0)	22 (16.5)	22 (16.5)	38 (28.6)	25 (18.8)	13 (9.8)

Treatment attribute, n (%)[a]	Very negative	Negative	Somewhat negative	Neutral	Somewhat positive	Positive	Very positive
Prucalopride	3 (2.4)	9 (7.1)	26 (20.6)	21 (16.7)	33 (26.2)	26 (20.6)	8 (6.3)
**Patient compliance**							
Linaclotide	0 (0.0)	1 (0.6)	7 (4.4)	12 (7.5)	45 (28.1)	64 (40.0)	31 (19.4)
Lubiprostone	0 (0.0)	4 (2.6)	12 (7.8)	20 (13.0)	50 (32.5)	55 (35.7)	13 (8.4)
Plecanatide	0 (0.0)	1 (0.8)	4 (3.0)	17 (12.8)	46 (34.6)	44 (33.1)	21 (15.8)
Prucalopride	1 (0.8)	0 (0.0)	7 (5.6)	22 (17.5)	37 (29.4)	42 (33.3)	17 (13.5)
**Patient satisfaction**							
Linaclotide	0 (0.0)	0 (0.0)	4 (2.5)	14 (8.8)	38 (23.8)	73 (45.6)	31 (19.4)
Lubiprostone	0 (0.0)	1 (0.6)	10 (6.5)	22 (14.3)	54 (35.1)	52 (33.8)	15 (9.7)
Plecanatide	0 (0.0)	0 (0.0)	2 (1.5)	13 (9.8)	50 (37.6)	50 (37.6)	18 (13.5)
Prucalopride	1 (0.8)	0 (0.0)	6 (4.8)	18 (14.3)	29 (23.0)	60 (47.6)	12 (9.5)
**Safety**							
Linaclotide	0 (0.0)	0 (0.0)	5 (3.1)	15 (9.4)	47 (29.4)	61 (38.1)	32 (20.0)
Lubiprostone	0 (0.0)	0 (0.0)	4 (2.6)	20 (13.0)	59 (38.3)	52 (33.8)	19 (12.3)
Plecanatide	0 (0.0)	0 (0.0)	3 (2.3)	17 (12.8)	44 (33.1)	49 (36.8)	20 (15.0)
Prucalopride	1 (0.8)	1 (0.8)	4 (3.2)	22 (17.5)	40 (31.7)	43 (34.1)	15 (11.9)

Data were collected via the HCP survey.

[a]Linaclotide (n = 160), lubiprostone (n = 154), plecanatide (n = 133) and prucalopride (n = 126).

CIC, chronic idiopathic constipation; HCP, health-care professional.

Supplementary Table 6. Patient satisfaction of four CIC prescription medications they were currently receiving: linaclotide, lubiprostone, plecanatide, and prucalopride

**Current prescription medication, n (%)[a]**	**Completely dissatisfied**	**Very dissatisfied**	**Somewhat dissatisfied**	**Neither satisfied nor dissatisfied**	**Somewhat satisfied**	**Very satisfied**	**Completely satisfied**
Linaclotide	0 (0.0)	1 (1.4)	3 (4.1)	4 (5.5)	16 (21.9)	39 (53.4)	10 (13.7)
Lubiprostone	0 (0.0)	0 (0.0)	0 (0.0)	0 (0.0)	6 (24.0)	15 (60.0)	4 (16.0)
Plecanatide	0 (0.0)	1 (3.4)	2 (6.9)	0 (0.0)	4 (13.8)	15 (51.7)	7 (24.1)
Prucalopride	0 (0.0)	0 (0.0)	1 (7.1)	1 (7.1)	4 (28.6)	6 (42.9)	2 (14.3)

Data were collected via the patient survey.

[a]Linaclotide (n = 73), lubiprostone (n = 25), plecanatide (n = 29) and prucalopride (n = 14).

CIC, chronic idiopathic constipation.

Supplementary Table 7. A list of the survey questions completed by the HCPs in this study

Survey question	Response
HCP demographics and patient management	
What is your primary specialty?	• General gastroenterologist<br>• Primary care physician/family practitioner<br>• Gastroenterologist motility specialist<br>• Nurse practitioner working in gastroenterology<br>• Nurse practitioner working in primary care<br>• Physician assistant working in gastroenterology<br>• Physician assistant working in primary care
In the last 12 months, approximately how many patients have you personally managed for CIC?	[Free text response]
How long have you been managing patients with CIC?	• Less than 1 year<br>• 1–3 years<br>• 3–5 years<br>• 5 years+
In what type of setting do you see patients?	• Community hospital<br>• Academic center<br>• Private center<br>• Government or VA hospital<br>• Outpatient clinic/office/family medicine center<br>• Long-term care facility or nursing home<br>• Other
In which of the following settings is your practice based?	• Urban<br>• Rural
Perceptions and expectations of medicine	
Importance in CIC management (respond for each of the following):<br>• aligns with treatment guidelines<br>• ease of access<br>• ease of treatment administration	• Not at all important<br>• Low importance<br>• Slightly important<br>• Neutral

• effective relief of pain<br>• effective relief of straining<br>• effective relief of symptoms<br>• familiarity/experience<br>• frequency of treatment administration<br>• improvement in sensations of incomplete evacuation<br>• improvement in stool consistency<br>• increased quality of life<br>• long-term efficacy<br>• low level of monitoring for mental health adverse events/ drug reactions<br>• low level of monitoring for physical adverse events/ drug reactions<br>• low level of patient support needed<br>• maintains patient’s ability to perform tasks/activities<br>• mode of treatment administration<br>• patient compliance<br>• rapid onset of action<br>• reasonable cost–benefit ratio<br>• reducing the chance of fecal impaction<br>• safe for short- and long-term use	• Moderately important<br>• Very important<br>• Extremely important
Barriers to treatment	
What, if anything, prevents you prescribing treatments to CIC patients (respond for each of the following):<br>• complexity of getting insurance coverage<br>• concerns with efficacy<br>• concerns with safety<br>• delays in diagnosis of CIC<br>• lack of evidence supporting their use<br>• lack of experience prescribing certain medications<br>• negative experience using prescription medications for previous patients<br>• patient did not want to take the medication prescribed to them	[0–100][a]

• patient out-of-pocket cost for medications<br>• patient preference for over-the-counter options<br>• patient reluctant to take any medications at all for their condition<br>• unaware of available treatment options<br>• other[b]	
Current treatment landscape	
In an ideal world, what would you choose to prescribe/recommend for each of the following lines of treatment?[c,d]<br>• First line<br>• Second line<br>• Third line<br>• Fourth line	• Bulk-forming laxatives<br>• Colchicine<br>• Dicyclomine hydrochloride<br>• Hyoscyamine<br>• Increased dietary fiber<br>• Increased hydration<br>• Increased physical activity<br>• Introducing a schedule for using the toilet<br>• Lactulose<br>• Linaclotide<br>• Lubiprostone<br>• Lubricant laxatives<br>• Misoprostol<br>• Osmotic laxatives<br>• Plecanatide<br>• Prucalopride<br>• Pyridostigmine<br>• Stimulant laxatives<br>• Tegaserod<br>• Tenapanor<br>• Using a toilet that is closer to the floor/adding a device to elevate the feet<br>• Using biofeedback methods
Perceptions and use of linaclotide, lubiprostone, plecanatide and prucalopride	

Of all your patients with CIC, what percentage: have never received prucalopride, but are clinically eligible	[Free text response]
Of all your patients with CIC, what percentage: have never received lubiprostone, but are clinically eligible	[Free text response]
Of all your patients with CIC, what percentage: have never received plecanatide, but are clinically eligible	[Free text response]
Of all your patients with CIC, what percentage: have never received linaclotide, but are clinically eligible	[Free text response]
Perception compared to other prescription medications (respond for each of the four prescription medications with each attribute):<br>• lubiprostone<br>• linaclotide<br>• plecanatide<br>• prucalopride<br><br>• access<br>• cost to patient (out-of-pocket costs)<br>• insurance coverage<br>• overall efficacy<br>• overall satisfaction<br>• patient compliance<br>• patient satisfaction<br>• safety<br>• your understanding/knowledge	• Very negative<br>• Negative<br>• Somewhat negative<br>• Neutral<br>• Somewhat positive<br>• Positive<br>• Very positive

[a]0 = issue does not prevent prescribing treatments at all; 100 = issue completely prevents prescribing a treatment; [b]There was no option in the survey for HCPs to provide further information; [c]HCPs could select more than one response; [d]Tegaserod and tenapanor are indicated for the treatment of irritable bowel syndrome with constipation in adult women aged < 65 years and in adults, respectively, dicyclomine hydrochloride is indicated for the treatment of functional bowel/irritable bowel syndrome in adults, and colchicine is indicated for the treatment of gout flares and Familial Mediterranean Fever in adults and children aged ≥ 4 years; hence, these prescription medications were excluded from these analyses.[1-4]

CIC, chronic idiopathic constipation; HCP, health-care professional; VA, Veterans Affairs.

Supplementary Table 8. A list of the survey questions completed by the patients in this study

**Survey question**	**Response**
Patient demographics	
Patient age	[Free text response]
Sex at birth	• Female<br>• Male<br>• Other<br>• Prefer not to say
Patient ethnicity	• American Indian, Indigenous American or Alaska Native<br>• Black or African American<br>• East or Southeast Asian<br>• Middle Eastern or North African<br>• Native Hawaiian or Pacific Islander<br>• South Asian (Indian subcontinent)<br>• White<br>• Other
Are you of Hispanic, Latin or Spanish origin?	• Yes<br>• No<br>• Don't know
How would you describe your work status?	• Full-time homemaker<br>• Full-time student<br>• Part-time student<br>• Retired<br>• Unable to work due to constipation<br>• Unemployed (for reasons related to condition<br>• Working full-time (in a paid job or as a volunteer)<br>• Working part-time (in a paid job or as a volunteer)<br>• Not listed
What best describes your type of health insurance coverage?[a]	• Cobra (continuation coverage)<br>• Employer provided/sponsored insurance

	• Health insurance exchange plan<br>• Medicaid (or equivalent in your state)<br>• Medicare<br>• Medicare advantage<br>• Medicare medical savings account (MSA)<br>• Medicare part D prescription drug plan<br>• Non-Medicare retired benefit<br>• Partner/family member employer<br>• Privately arranged insurance<br>• Tricare/Veterans' health care<br>• No insurance coverage
Treatment journey and perception	
Which, if any, of the below behavioral/lifestyle modifications are you currently using to treat constipation?[a]	• Drinking more water<br>• Exercising more<br>• Introducing a schedule for using the toilet<br>• Taking in more dietary fiber<br>• Using a toilet that is closer to the floor/adding a device to elevate<br>your feet<br>• Using biofeedback therapy<br>• None of the above
How effective were behavioral/lifestyle modifications in relieving the symptoms of constipation (respond for each of the following):<br>• drinking more water<br>• exercising more<br>• introducing a schedule for using the toilet<br>• taking in more dietary fiber<br>• using a toilet that is closer to the floor/adding a device to elevate your feet<br>• using biofeedback therapy	• Not all effective<br>• Low effectivity<br>• Slightly effective<br>• Neutral<br>• Moderately effective<br>• Very effective<br>• Extremely effective<br>• N/A
What prescription medications are you currently receiving for constipation?[a,b]	• Colchicine<br>• Dicyclomine hydrochloride<br>• Hyoscyamine

	• Lactulose<br>• Linaclotide<br>• Lubiprostone<br>• Misoprostol<br>• Plecanatide<br>• Prucalopride<br>• Pyridostigmine<br>• Tegaserod<br>• Tenapanor
How important is each attribute to you when thinking about the treatment of your constipation (respond for each of the following):<br>• health-care professional recommendation based on their patient's previous experience<br>• treatment doesn't have many side effects<br>• treatment is affordable<br>• treatment is easy to take<br>• treatment is safe to use (short and long term)<br>• works well in relieving symptoms (including abdominal bloating and discomfort)	• Extremely unimportant<br>• Very unimportant<br>• Somewhat unimportant<br>• Neutral<br>• Somewhat important<br>• Very important<br>• Extremely important
Which, if any, of the below have you experienced in relation to your medications/treatment to treat your constipation[a]	• Couldn't afford an appointment with a health-care provider<br>• Difficulty getting an appointment to see a health-care provider about treatment for your constipation<br>• Having to travel a long way to see a health-care provider for your constipation, meaning you see your health-care practitioner less than you should<br>• Issues accessing online/telemedicine appointments and weren't able to attend face-to-face appointments with a HCP due to COVID restrictions<br>• The medication that the health-care provider wanted to prescribe would mean you had to pay too much, so you did not take the medication<br>• The travel to the health-care provider is expensive, meaning you see your health-care practitioner less than you should

	• You did not realize that prescription treatments were available for constipation<br>• Your HCP did not know about prescription treatment options<br>• Your HCP was reluctant to prescribe a medication<br>• Your insurance did not cover your prescription medication for your constipation
The last 30 days, how much (on average) did you pay towards the expenses specifically relating to your constipation (respond for each of the following):<br>• HCP appointments<br>• co-payment for prescription medications<br>• OTC treatments<br>• transport to medical appointments<br>• tests or laboratory results<br>• emergency department visits<br>• parking fees	[Free text response]
How satisfied, if at all, are you with the current treatment you are receiving for your constipation (respond for each of the following):<br>• linaclotide<br>• lubiprostone<br>• plecanatide<br>• prucalopride	• Completely dissatisfied<br>• Very dissatisfied<br>• Somewhat dissatisfied<br>• Neither satisfied nor dissatisfied<br>• Somewhat satisfied<br>• Very satisfied<br>• Completely satisfied

[a]Patients could select more than one response; [b]Tegaserod and tenapanor are indicated for the treatment of irritable bowel syndrome with constipation in adult women aged < 65 years and in adults, respectively, dicyclomine hydrochloride is indicated for the treatment of functional bowel/irritable bowel syndrome in adults, and colchicine is indicated for the treatment of gout flares and Familial Mediterranean Fever in adults and children aged ≥ 4 years; hence, these prescription medications were excluded from these analyses.[1-4]

CIC, chronic idiopathic constipation; HCP, health-care professional; N/A, not applicable; OTC, over-the-counter.

Supplementary Table 9. First-line CIC treatments received by patients, overall and by HCP specialty

Treatment, n (%)[a]	Total (N = 303)	General gastroenterologist (n = 74)	Motility specialist (n = 30)	Primary care physician (n = 87)	Advanced practice provider (n = 112)
Bulk-forming laxatives	136 (44.9)	35 (47.3)	16 (53.3)	37 (42.5)	48 (42.9)
Osmotic laxatives	134 (44.2)	28 (37.8)	17 (56.7)	44 (50.6)	45 (40.2)
Stimulant laxatives	110 (36.3)	33 (44.6)	13 (43.3)	27 (31.0)	37 (33.0)
Linaclotide	69 (22.8)	22 (29.7)	2 (6.7)	24 (27.6)	21 (18.8)
Lubiprostone	58 (19.1)	18 (24.3)	9 (30.0)	15 (17.2)	16 (14.3)
Lubricant laxatives	44 (14.5)	11 (14.9)	9 (30.0)	12 (13.8)	12 (10.7)
Plecanatide	34 (11.2)	9 (12.2)	2 (6.7)	12 (13.8)	11 (9.8)
Prescription osmotic laxatives	25 (8.3)	3 (4.1)	3 (10.0)	13 (14.9)	6 (5.4)
Prucalopride	18 (5.9)	7 (9.5)	3 (10.0)	4 (4.6)	4 (3.6)
Hyoscyamine	13 (4.3)	1 (1.4)	0 (0.0)	7 (8.0)	5 (4.5)
Misoprostol	2 (0.7)	1 (1.4)	0 (0.0)	1 (1.1)	0 (0.0)
Pyridostigmine	2 (0.7)	2 (2.7)	0 (0.0)	0 (0.0)	0 (0.0)

Data were collected via the CRF. For each line of treatment, HCPs could select more than one treatment. Data are ordered by total frequency.
[a]Tegaserod and tenapanor are indicated for the treatment of irritable bowel syndrome with constipation in adult women aged < 65 years and in adults, respectively, dicyclomine hydrochloride is indicated for the treatment of functional bowel/irritable bowel syndrome in adults, and colchicine is indicated for the treatment of gout flares and Familial Mediterranean Fever in adults and children aged ≥ 4 years; hence, these prescription medications were excluded from this table.[1-4]
CIC, chronic idiopathic constipation; CRF, case report form; HCP, health-care professional.

Supplementary Table 10. Direct and indirect expenses associated with CIC treatment reported by patients for the past 30 days

Expense in the past 30 days (N = 230)	Cost, $, mean (SD)
HCP appointments (n = 178)	30.3 (49.4)
Co-payment for prescription medications (n = 187)	28.4 (40.0)
OTC treatments (n = 199)	24.3 (20.3)
Transport to medical appointments (n = 179)	23.5 (32.7)
Tests or laboratory results (n = 158)	22.1 (51.7)
Emergency department visits (n = 154)	7.6 (54.0)
Parking fees (n = 162)	4.1 (10.8)

Data were collected via the patient survey.

HCP, health-care professional; OTC, over-the-counter; SD, standard deviation.

References

1. US WorldMeds. ZELNORM (tegaserod). Highlights of prescribing information. www.accessdata.fda.gov/drugsatfda_docs/label/2019/021200Orig1s015lbl.pdf. Accessed March 9, 2026
2. Ardelyx, Inc. IBSRELA (tenapanor). Highlights of prescribing information. www.accessdata.fda.gov/drugsatfda_docs/label/2019/211801s000lbl.pdf. Accessed March 9, 2026
3. AXCAN Pharma US, Inc. BENTYL (dicyclomine hydrochloride). Highlights of prescribing information. https://www.accessdata.fda.gov/drugsatfda_docs/label/2011/007409s041lbl.pdf. Accessed March 9, 2026
4. Mutual Pharmaceutical Company, Inc. COLCRYS (colchicine). Highlights of prescribing information. . https://www.accessdata.fda.gov/drugsatfda_docs/label/2009/022351lbl.pdf. Accessed March 9, 2026
