# Supplementary material for: Treatment Patterns, Perceptions, Barriers, and Costs in Patients With Chronic Idiopathic Constipation in the United States
Source: Gastro Hep Adv. 2026 Feb 17;5(4):100900. doi: 10.1016/j.gastha.2026.100900 (PMC13018922; doi:10.1016/j.gastha.2026.100900)
Supplement: Extended PDF [file mmc2.pdf]

Gastro Hep Advances 2026;5:100900

ORIGINAL RESEARCH—CLINICAL

Treatment Patterns, Perceptions, Barriers, and Costs in Patients With Chronic Idiopathic Constipation in the United States

Darren M. Brenner,[1,*] Baharak Moshiree,[2,*] Joanna de Courcy,[3] Neil Reynolds,[3] Teresa Taylor-Whiteley,[3] Jeanne Jiang,[4] Mei Lu,[4] Brian Terreri,[4] and Eric D. Shah[5]

[1]Feinberg School of Medicine, Northwestern University, Chicago, Illinois; [2]Atrium Health, Wake Forest University School of Medicine, Charlotte, North Carolina; [3]Adelphi Real World, Bollington, UK; [4]Takeda Pharmaceuticals USA, Inc, Lexington, Massachusetts; and [5]Division of Gastroenterology and Hepatology, University of Michigan, Ann Arbor, Michigan

BACKGROUND AND AIMS: Limited real-world data are available on barriers to prescribing or recommending treatments for chronic idiopathic constipation (CIC), treatment experiences and expectations, and the financial impact of CIC from the perspectives of patients and health-care professionals (HCPs). **METHODS:** In this noninterventional, cross-sectional, retrospective survey in the United States (October 2022–June 2023), board-certified gastroenterologists, motility specialists, advanced practice providers, and primary care physicians each recruited up to 8 adults with HCP-diagnosed CIC and no previous CIC clinical trial enrollment. HCP and patient surveys, and case report forms (CRFs) captured demographics and treatment patterns, perceptions, barriers, and costs. **RESULTS:** Overall, 170 HCPs completed CRFs for 368 patients, of whom 230 completed the patient survey. Mean (standard deviation) patient age was 50.2 (16.5) years (CRF) and 49.7 (16.2) years (patient survey). HCPs would ideally recommend lifestyle/dietary modifications and over-the-counter treatments before prescription medications. Most HCPs rated increased quality of life (73.5%) and long-term efficacy (71.2%) as important when managing CIC, whereas most patients considered symptom relief (83.8%) and affordability (80.7%) as important. HCPs did not report substantial barriers to prescribing treatments, whereas patients reported difficulty getting an HCP appointment (36.3%) and a lack of awareness of CIC prescription medications (34.8%). Most HCPs and patients were satisfied with treatment options. The greatest patient expenses (30-day mean costs [$]) were seeing an HCP ($30.3) and prescription medication co-payments ($28.4). **CONCLUSION:** Despite most patients feeling satisfied with CIC treatment options, education on the availability of prescription medication in the United States is needed. This limited awareness, the patient expense of HCP visits, and prescription medication co-payments may limit patient access to CIC therapies.

Keywords: Chronic Idiopathic Constipation; Treatment Patterns; Perceptions; Barriers; Costs

Introduction

Chronic idiopathic constipation (CIC), or functional constipation, is a common disorder of gut–brain interaction with no underlying physiological cause.[1] Diagnosis is typically based on reports of difficult, infrequent, or incomplete defecation and/or hard stools, as classified by the Rome IV criteria.[2,3] The prevalence of CIC in the United States is estimated to range from 9% to 20%.[4] Prevalence is higher among women and patients with low socioeconomic status compared with men and patients with high socioeconomic status, respectively, and generally increases with age.[1] Patients with CIC have reduced health-related quality of life and greater direct health-care costs than the general US population.[5,6] Recent literature on the economic burden of CIC is limited; however, in 2010, average all-cause health-care costs for patients with CIC were estimated to be $9012 per person per year,[7] and current costs are predicted to be higher.

An online US questionnaire completed by patients with CIC in 2017[8] indicated that most patients managed their disease with lifestyle changes, such as increased fiber consumption and/or over-the-counter (OTC) laxatives, before consulting a health-care professional (HCP). This strategy aligns with the current clinical guidelines from the American Gastroenterological Association (2023) for the management of CIC[9]; however, data suggest only 40% of patients with CIC are satisfied with OTC treatments.[8] In patients who do not respond or are intolerant to OTC treatments, the guidelines recommend the use of pharmacologic agents or prescription medications.[9] Four prescription medications are now approved by the US Food and Drug Administration (FDA) specifically for the treatment of adults with CIC (linaclotide, lubiprostone, plecanatide, and prucalopride).[10–13] The efficacy and safety of these prescription medications in adult

*Denotes co-first authorship.

Abbreviations used in this paper: CIC, chronic idiopathic constipation; CRF, case report form; FDA, US Food and Drug Administration; HCP, health-care professional; OTC, over-the-counter; SD, standard deviation.

Most current article

Copyright © 2026 The Authors. Published by Elsevier Inc. on behalf of the AGA Institute. This is an open access article under the CC BY license (http://creativecommons.org/licenses/by/4.0/).
2772-5723
https://doi.org/10.1016/j.gastha.2026.100900

patients with CIC has been well established in double-blind, placebo-controlled, randomized clinical studies.[14–17] However, published data on the use of these therapies in real-world clinical practice are limited.

This study aimed to identify potential real-world barriers to prescribing or recommending treatments for CIC, to examine perceptions and expectations of these treatments, and to explore the financial impact of CIC on patients, from the perspectives of patients with CIC and their treating HCPs.

Methods

Study Design

This noninterventional, cross-sectional, retrospective survey was conducted in the United States between October 2022 and June 2023 and recruited adult patients with CIC and HCPs with patients with CIC in their care. The study design is presented in Supplementary Figure 1.

HCPs were eligible for enrollment in this study if they were board-certified gastroenterologists, motility specialists, advanced practice providers, or primary care physicians currently practicing in the United States, had made treatment decisions for more than 5 patients with CIC, had managed more than 10 patients with CIC in the past year, had more than 3 years' experience in managing patients with CIC, and had access to patient medical records. Eligible HCPs were recruited via Survey Healthcare Globus and completed a 15-minute survey. This survey included questions on their demographics, treatment and prescribing patterns, perceptions and expectations of available CIC medications, and perceived treatment barriers.

Each HCP recruited up to 8 of the patients with whom they next consulted and who met the eligibility criteria. Patients were eligible for inclusion if they were aged at least 18 years, had an HCP diagnosis of CIC, could understand and provide informed consent, and were not previously nor currently enrolled in a clinical trial for CIC. Enrolled patients were asked to complete a 30-minute survey which included questions on their demographics, clinical status, experiences of the diagnostic and management journey, treatments, treatment barriers, perceptions of treatment attributes and effectiveness of CIC treatments, and patient expenses. For each patient, a case report form (CRF) was completed by their HCP (not all patients included in the CRF analysis completed the patient survey). The CRF included clinical information on patient demographics, clinical status, diagnostic journey, and treatment history. The risk of information and recall bias was mitigated by matching the patient survey to the CRF completed by the HCP.

Study Outcomes

HCP and patient demographics. HCP demographics were captured in the HCP survey, and patient demographics were captured in the CRFs, both of which were completed by HCPs. A subset of the included patients also completed the patient survey; these patient demographics are reported separately.

Treatments for CIC. HCPs selected the lifestyle changes, OTC treatments, and prescription medications that they would be most likely to recommend in an ideal world as first-, second-, third-, and fourth-line treatments to patients with CIC from a list of 22 prespecified options (reported in Supplementary Table 1). HCPs also rated 22 CIC treatment attributes (including relief of symptoms, level of support and monitoring required, patient burden, cost, and safety) according to their importance in the treatment of their patients with CIC on a 7-point scale (Supplementary Table 2). Similarly, patients rated 6 attributes (affordability, ease of treatment administration, effectiveness in symptom relief, HCP recommendation, side effects, and short- and long-term safety) according to importance on a 7-point scale (Supplementary Table 3). In addition, HCPs recorded the first-, second-, third-, and fourth-line treatments being received by patients with CIC at the time of the study in the CRFs.

Treatment barriers. HCPs rated the most common barriers to prescribing CIC medications by allocating up to 100 points to each of the 13 attributes (0 = issue does not prevent prescribing treatments at all and 100 = issue completely prevents prescribing a treatment). Patients reported the most common barriers to receiving CIC treatments from a list of 10 prespecified scenarios (patient survey) and selected any that they had experienced in relation to their treatments for CIC.

HCP and patient perceptions of CIC prescription medication. HCPs and patients reported their perceptions of the effectiveness of 6 prespecified lifestyle or dietary modifications for relieving symptoms of CIC by ranking them using a 7-point scale (Supplementary Table 4). HCPs also reported their perceptions of 4 prescription medications for CIC: linaclotide, lubiprostone, plecanatide, and prucalopride. Nine attributes (overall satisfaction, access, cost, efficacy, HCP knowledge/understanding, insurance coverage, patient compliance, patient satisfaction, and safety) were rated on a 7-point scale (Supplementary Table 5). Patients also reported their overall satisfaction with current use of these same 4 prescription medications using a 7-point scale (Supplementary Table 6). Finally, patients reported their satisfaction with the treatments they were currently receiving or had previously taken for CIC. HCPs reported perceived patient satisfaction with the current or previously received CIC treatments in the CRF.

Financial impact of CIC. Patients reported direct and indirect expenses specifically related to CIC over the past 30 days for 7 prespecified potential sources of expenses. All costs are reported as US dollars.

A full list of survey questions presented to the HCPs and patients in this study are provided in Supplementary Tables 7 and 8, respectively.

Data Analysis

Descriptive analyses (ie, mean [standard deviation, (SD)] or n [%]) were conducted using IBM Survey Reporter (version 7.5). No statistical testing was performed for the analyses reported. The risk of missing data was minimized by the inclusion of a progress bar in the online HCP survey so that HCPs could track their progress and completion of the survey. For the patient survey, short and concise instructions were provided, along with minimized logic, which helped to ensure that patients understood each question.

Results

HCP Demographics

In total, the survey was sent to 42,215 HCPs, of whom 361 were screened out and 170 completed responses for

analysis. Most were primary care physicians/family practitioners (37.6% [64/170]) or general gastroenterologists (31.2% [53/170]). Most HCPs had more than 5 years' experience in managing patients with CIC (86.5% [147/170]), and on average, HCPs had managed 214.5 patients with CIC in the past 12 months. Most HCPs were based in an urban practice (79.4% [135/170]) (Table 1).

Table 1. HCP Demographics and Characteristics

Demographic/characteristic	HCPs (N = 170)
Primary specialty, n (%)	
Primary care physician/family practitioner	64 (37.6)
General gastroenterologist	53 (31.2)
Nurse practitioner working in gastroenterology	16 (9.4)
Gastroenterologist motility specialist	12 (7.1)
Nurse practitioner working in primary care	12 (7.1)
Physician assistant working in primary care	7 (4.1)
Physician assistant working in gastroenterology	6 (3.5)
Number of patients with CIC managed over the past 12 mo, mean (SD)	214.5 (239.6)
Time spent managing patients with CIC, n (%)	
Fewer than 3 y	0 (0.0)
3–5 y	23 (13.5)
More than 5 y	147 (86.5)
Practice setting, n (%)	
Outpatient clinic/office/family medicine center	73 (42.9)
Community hospital	35 (20.6)
Academic center	18 (10.6)
Private center	12 (7.1)
Government or Veterans Affairs hospital	1 (0.6)
Long-term care facility or nursing home	1 (0.6)
Other	30 (17.6)
Practice location, n (%)	
Urban	135 (79.4)
Rural	35 (20.6)

Data were collected via the HCP survey.

Patient Demographics

Overall, HCPs completed CRFs for 368 patients; of those, 230 patients completed the patient survey. The mean (SD) age of patients from the CRF and patients who completed the survey was 50.2 (16.5) years and 49.7 (16.2) years, respectively; most patients were female (CRF, 61.1% [225/368]; patient survey, 64.3% [148/230]). Most patients were White (CRF, 64.1% [236/368]; patient survey, 68.3% [157/230]) or Black or African American (CRF, 21.2% [78/368]; patient survey, 16.1% [37/230]). Overall, 13.6% (50/368) and 14.3% (33/230) of patients included in the CRF and those who completed the survey, respectively, were of Hispanic, Latin, or Spanish origin. Patients generally worked full time (CRF: 50.8% [187/368]; patient survey: 54.3% [125/230]) and had employer-provided/sponsored (52.3% [114/218]) or Medicare/Medicaid health insurance coverage (32.1% [70/218]) (patient survey). The demographics of the CRF patient population and those patients who completed the survey were generally similar (Table 2).

Treatments for CIC

In an ideal world, HCPs were most likely to recommend increased dietary fiber (88.8% [151/170]), increased hydration (81.8% [139/170]), and increased physical activity (78.2% [133/170]) as first-line treatments and osmotic laxatives (51.2% [87/170]), bulk-forming laxatives (41.8% [71/170]), and stimulant laxatives (40.0% [68/170]) as second-line treatments (Figure 1). Overall, first- and second-line treatment recommendations were generally similar across HCP specialties, although general gastroenterologists and motility specialists were more likely to recommend prescription medications as a second-line treatment compared with other HCPs (Supplementary Table 1). Overall, HCPs were most likely to recommend prescription medications as third- and fourth-line treatments; however, this was generally driven by the prescribing habits of the advanced practice providers (Figure 1 and Supplementary Table 1).

Of the 368 patients included in the CRF, 303 (82.3%) patients were receiving a first-line treatment for CIC, most commonly bulk-forming laxatives (44.9% [136/303]), osmotic laxatives (44.2% [134/303]), and/or stimulant laxatives (36.3% [110/303]). Of these 303 patients, 201 (66.3%) were taking prescription medication as a first-line treatment, most commonly linaclotide (22.8% [69/303]) and lubiprostone (19.1% [58/303]). Overall, linaclotide and lubiprostone were also the most common first-line prescription medications provided across all HCP specialties, with the exception of motility specialists where prucalopride was the second most common prescription medication at first-line (Supplementary Table 9). In addition, HCPs reported that in patients who had never received the following prescription medications (linaclotide, lubiprostone, plecanatide, and prucalopride), a mean percentage of 37.0%–42.2% were clinically eligible (as determined by each HCP).

When considering the management of patients with CIC, most HCPs rated increased quality of life (73.5% [125/170]), long-term efficacy (71.2% [121/170]), effective relief of symptoms (70.6% [120/170]), safe for short- and long-term use (68.8% [117/170]), effective relief of pain (67.6% [115/170]), patient compliance (64.7% [110/170]), and ease of access (62.4% [106/170]) as 'very' or 'extremely' important (Figure 2 and Supplementary Table 2). In the patient survey, symptom relief (83.8% [192/229]), affordability (80.7% [184/228]), and short- and long-term safety (77.3% [177/229]) were most frequently rated as 'very' or 'extremely' important when choosing a treatment for CIC (Supplementary Table 3).

Table 2. Patient Demographics and Characteristics

Demographic/Characteristic	All Patients (CRF) (N = 368)	Survey Respondents (N = 230)[a]
Age, y, mean (SD)	50.2 (16.5)	49.7 (16.2)
Sex, n (%)		
Female	225 (61.1)	148 (64.3)
Male	141 (38.3)	82 (35.7)
Not reported	2 (0.5)	0 (0.0)
BMI, kg/m^2, mean (SD)	26.9 (4.9)	–
Race/ethnicity, n (%)[b]		
White	236 (64.1)	157 (68.3)
Black or African American	78 (21.2)	37 (16.1)
South Asian (Indian subcontinent)	20 (5.4)	16 (7.0)
East or Southeast Asian	12 (3.3)	6 (2.6)
American Indian, Indigenous American, or Alaska Native	6 (1.6)	0 (0.0)
Middle Eastern or North African	5 (1.4)	3 (1.3)
Native Hawaiian or Pacific Islander	1 (0.3)	0 (0.0)
Other	15 (4.1)	22 (9.6)
Ethnicity, n (%)		
Hispanic, Latin, or Spanish origin	50 (13.6)	33 (14.3)
Employment/work status, n (%)		
Working full time	187 (50.8)	125 (54.3)
Retired	62 (16.8)	47 (20.4)
Working part time	53 (14.4)	26 (11.3)
Homemaker	24 (6.5)	15 (6.5)[c]
Unemployed	15 (4.1)	5 (2.2)[d]
Student	9 (2.4)	6 (2.6)[e]
On long-term sick leave	4 (1.1)	–
Not reported	14 (3.8)	6 (2.6)
Health insurance coverage, n (%)[f]		
Employer-provided/sponsored	–	114 (52.3)[g]
Medicare/Medicaid	–	70 (32.1)[h]
Privately arranged	–	23 (10.6)
Other	–	5 (2.3)[i]
None	–	6 (2.8)
No answer provided	–	12 (5.5)

Data were collected via the CRF or the patient survey.
BMI, body mass index.
[a]Not all patients included in the CRF analysis completed the patient survey.
[b]More than one response option could be selected.
[c]Full-time homemakers.
[d]Unemployed for reasons related to CIC.
[e]Full-time student, n = 5 (2.2%); part-time student, n = 1 (0.4%).
[f]N = 218.
[g]Via patient's employer, n = 91 (41.7%); via partner/family's employer, n = 23 (10.6%).
[h]Medicare, n = 28 (12.8%); Medicaid (or equivalent in state), n = 23 (10.6%); Medicare Advantage, n = 10 (4.6%); Medicare Part D prescription drug plan, n = 9 (4.1%).
[i]Health insurance exchange plan, n = 4 (1.8%); TRICARE/Veterans Health Care, n = 1 (0.5%).

Treatment Barriers

Overall, HCPs did not report having substantial difficulties in prescribing treatments for CIC. HCPs found patient out-of-pocket costs (mean [SD] score out of 100: 18.8 [18.0]) and the complexity of getting insurance coverage (17.3 [16.2]) to be the greatest barriers; patient preference for OTC treatments (8.2 [8.9]) also made prescribing treatments difficult for HCPs. The barriers most commonly experienced by patients were difficulty getting an HCP appointment (36.3% [49/135]), lack of awareness that prescription medications were available for CIC (34.8% [47/135]), and unavailability of insurance for the CIC prescription medication (25.9% [35/135]) (Table 3).

HCP and Patient Perceptions of CIC Treatments

Most patients were using a lifestyle change to treat their CIC at the time of study. The most common included drinking more water (86.3% [189/219]), increasing dietary fiber (80.8% [177/219]), and exercise (63.9% [140/219]). Overall, higher proportions of HCPs than patients reported lifestyle changes as effective in relieving the symptoms of CIC (except for a toilet that is closer to the floor/elevation

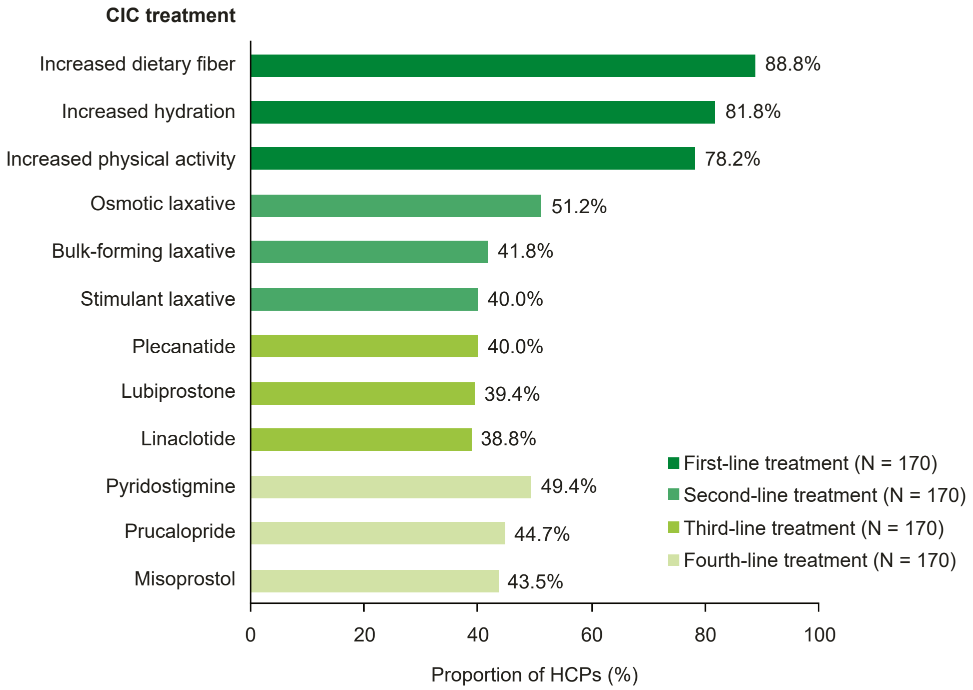

Figure 1. The 3 most commonly reported treatments that HCPs would recommend in an ideal world for patients with CIC for first-, second-, third-, and fourth-line treatments. Data were collected via the HCP survey. For each line of treatment, HCPs could select more than 1 treatment from 22 options: lifestyle or dietary modifications (increased dietary fiber, increased hydration, increased physical activity, introducing a schedule for using the toilet [habit training], using a toilet that is closer to the floor or adding a device to elevate the feet, using biofeedback methods); over-the-counter medications (bulk-forming laxatives, lactulose [generics], lubricant laxatives, osmotic laxatives, stimulant laxatives); prescription medications (colchicine [generics, Colcrys, Gloperba, Mitigare], dicyclomine hydrochloride [generics, Bentyl][a], hyoscyamine [Levsin], linaclotide [Linzess], lubiprostone [generic, Amitiza], misoprostol [generics, Cytotec], plecanatide [Trulance], prucalopride [Motegrity], pyridostigmine [Mestinon], tegaserod [Zelnorm][a], tenapanor [Ibsrela][a]). The top 3 treatments for each treatment line are shown. [a]Tegaserod and tenapanor are indicated for the treatment of irritable bowel syndrome with constipation in adult women aged $<$65 years and in adults, respectively, dicyclomine hydrochloride is indicated for the treatment of functional bowel/irritable bowel syndrome in adults, and colchicine is indicated for the treatment of gout flares and Familial Mediterranean Fever in adults and children aged $\geq$4 years; hence, these prescription medications were excluded from this table.[18–21]

device) (Figure 3). Of the 6 lifestyle changes assessed for CIC, increased hydration was considered effective the most frequently by HCPs and patients (65.9% [207/314] of HCPs rated this as ‘somewhat’ to ‘extremely’ effective; 57.4% [124/216] of patients rated this as ‘moderately’ to ‘extremely’ effective). A toilet schedule was considered effective the least frequently (47.8% [64/134] of HCPs rated this as ‘somewhat’ to ‘extremely’ effective; 37.7% [29/77] of patients rated this as ‘moderately’ to ‘extremely’ effective) (Figure 3 and Supplementary Table 4).

Regarding the 4 prescription medications for CIC, a higher proportion of HCPs rated linaclotide (63.8% [102/160]) as ‘positive’ and ‘very positive’ in terms of their overall satisfaction in comparison with prucalopride (51.6% [65/126]), lubiprostone (47.4% [73/154]), and plecanatide (47.4% [63/133]). Findings were similar for the following attributes: access, cost to patient (out-of-pocket costs), efficacy, HCP knowledge/understanding, insurance coverage, patient compliance, patient satisfaction, and safety (Figure 4 and Supplementary Table 5). Most patients were satisfied with the 4 prescription medications examined, with a slightly higher proportion of patients ‘somewhat’ to ‘completely’ satisfied with lubiprostone (100.0% [25/25]) than with plecanatide (89.7% [26/29]), linaclotide (89.0% [65/73]), and prucalopride (85.7% [12/14]) (Supplementary Table 6).

Financial Impact of CIC

In the 30 days before the study, the greatest patient expenses (mean [SD]) were related to seeing an HCP ($30.3 [$49.4]), co-payment for prescription medications ($28.4 [$40.0]), and OTC treatments ($24.3 [$20.3]) (Supplementary Table 10). Patients insured by Medicare experienced nearly double the cost of OTC treatments ($27.3 [$18.6], n = 26 vs $11.2 [$13.1], n = 20, respectively) and prescription medications ($20.9 [$43.0], n = 17 vs $11.8 [$17.9], n = 19), respectively) than those insured by Medicaid; similar trends were observed for Medicare Part D and Medicare Advantage. Patients with employer-provided insurance (n = 86) reported a 30-day cost for prescription medications of $34.7 ($48.7). The cost for

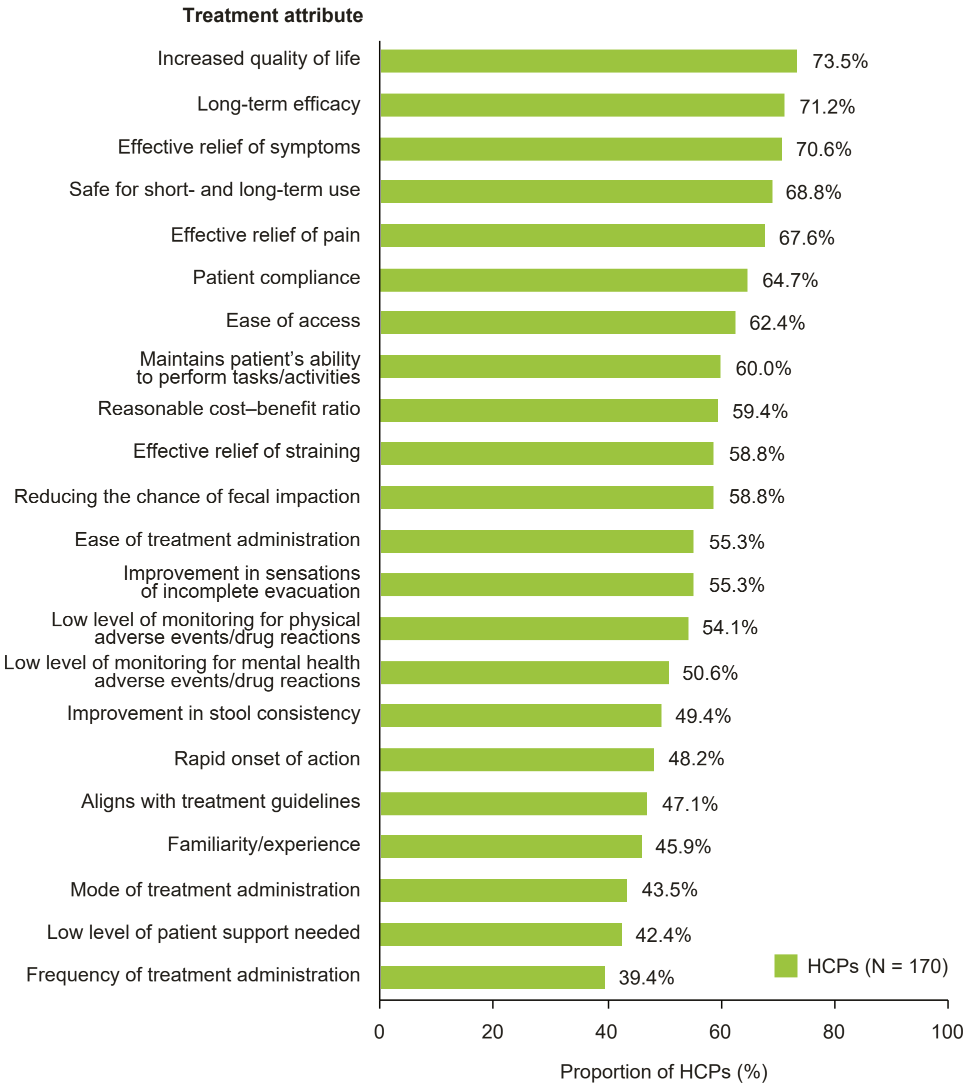

Figure 2. Proportions of HCPs who considered a CIC treatment attribute important when managing CIC. Data were collected via the HCP survey. For each option, HCPs could choose a rating from a 7-point scale: 'not at all important,' 'low importance,' 'slightly important,' 'neutral,' 'moderately important,' 'very important,' and 'extremely important.' Data are presented for attributes rated as 'very important' or 'extremely important.'

seeing an HCP with Medicare and Medicaid were similar ($13.3 [$18.2], n = 18 vs $12.4 [$27.3], n = 18, respectively).

Discussion

There are limited real-world data available on patients' and HCPs' experiences and perceptions of the CIC treatment landscape, the barriers to receiving treatment for CIC, and up-to-date associated health-care costs. When asked what they would recommend for patients with CIC in an ideal world, HCPs reported that they would initially recommend lifestyle or dietary modifications, then OTC treatments, which would subsequently be escalated to prescription medications. Overall, most patients and HCPs were satisfied with the treatment options available for CIC, although there were some distinct differences between patient and HCP perceptions of the importance of specific treatment attributes in disease management. Most HCPs rated increased quality of life and long-term efficacy as the important attributes in the management of patients with CIC, whereas patients considered symptom relief and treatment affordability as the most important attributes when choosing a treatment for CIC. Out-of-pocket costs and difficulty obtaining insurance coverage were reported as the most common barriers to prescribing CIC medications by HCPs, but patients reported difficulty getting an HCP appointment and lack of awareness of prescription medications for CIC.

The HCPs in this study were generally found to follow current treatment guidelines for CIC[9] by initially recommending lifestyle modifications as first-line treatment options and then escalating to OTC treatments. Despite insufficient evidence in the literature,[9,22] more than 80% of HCPs would recommend increased hydration as a first-line treatment for CIC. Overall, a higher proportion of HCPs than patients reported lifestyle modifications as effective in relieving symptoms of CIC, including consumption of dietary fiber and increased hydration. These data indicate discordance between HCP and patient perceptions of the benefits of lifestyle modifications and suggest that such treatments may not be effective for all patients. There is the possibility, however, of a selection bias in this consulting population

Table 3. Experiences of Barriers to Treatment for CIC as Rated by HCPs and Patients

Barrier to Treatment	Experience
HCPs (N = 170)	Score out of 100, mean (SD)
Patient out-of-pocket cost for medications	18.8 (18.0)
Complexity of getting insurance coverage	17.3 (16.2)
Patient preference for over-the-counter options	8.2 (8.9)
Patient reluctant to take any medications at all for their condition	7.6 (10.7)
Concerns with efficacy	7.6 (9.8)
Concerns with safety	7.2 (9.9)
Patient did not want to take the medication prescribed to them	6.7 (7.5)
Lack of experience prescribing certain medications	6.2 (8.1)
Delays in diagnosis of CIC	5.3 (6.8)
Lack of evidence supporting their use	4.9 (7.4)
Negative experience using prescription medications for previous patients	4.9 (5.7)
Unaware of available treatment options	4.3 (6.8)
Other[a]	1.1 (3.0)
Patients (N = 135)	Proportion of Patients, n (%)
Difficulty getting an HCP appointment	49 (36.3)
Not aware that prescription medications were available for CIC	47 (34.8)
CIC prescription medication was not covered by insurance	35 (25.9)
Having to travel a long way to see an HCP	26 (19.3)
Out-of-pocket costs for the prescription medication too high	15 (11.1)
HCP appointment too expensive	12 (8.9)
Issues accessing online/telemedicine appointments and not being able to attend face-to-face appointments with an HCP due to COVID restrictions	12 (8.9)
Travel to see an HCP is expensive	8 (5.9)
HCP was reluctant to prescribe a medication	4 (3.0)
HCP did not know about the prescription treatment options	3 (2.2)

Data were collected via the HCP survey and the patient survey. The HCP survey collected data for each barrier using a 100-point scoring system: 0 = the issue does not prevent prescribing treatments at all; 100 = the issue completely prevents prescribing treatments. The patient survey collected data for 10 scenarios by patients selecting those that they had experienced.
COVID, coronavirus disease.
[a]There was no option in the survey for HCPs to provide further information.

because patients who find dietary and lifestyle modifications effective may be less likely to consult with their HCP.

In an ideal world, HCPs reported that they would escalate to prescription medications as third- and fourth-line interventions. However, the responses from the HCPs suggested that approximately 40% of the patients who had never received prescription medication for CIC were clinically eligible to do so (as determined by each HCP). Although this could be attributed to successful treatment with nonprescription therapies, this could also indicate wider accessibility issues. A lack of awareness of available prescription medications was identified as the most common barrier to treatment by patients, suggesting that further patient and HCP education would be beneficial. In addition, HCPs identified the complexity of obtaining insurance coverage as a common barrier to prescribing CIC medications, and a 2021 study found that the optimal cost-effective CIC treatment algorithm from the insurer's perspective was to deny access to prescription drugs regardless of whether patients failed OTC treatments.[23] The cost for co-payment for prescription medications was also identified in our study as one of the greatest expenses for patients with CIC. These data suggest that cost should be addressed at a policy level to ensure that patients have access to optimal treatments.

When comparing the 4 CIC prescription medications in terms of overall satisfaction, HCPs rated linaclotide slightly more positively than lubiprostone, plecanatide, and prucalopride. Linaclotide was approved by the US FDA for CIC in 2012,[10] whereas plecanatide and prucalopride are newer drugs to the US market, having received FDA approval in 2017 and 2018, respectively.[12,13] It is therefore possible that HCPs do not have the extent of knowledge of and experience with plecanatide and prucalopride that they do with linaclotide and thus may be less likely to prescribe these medications. These data could also suggest linaclotide is more easily accessible than the newly approved medications, and access could be restricted by step therapies.

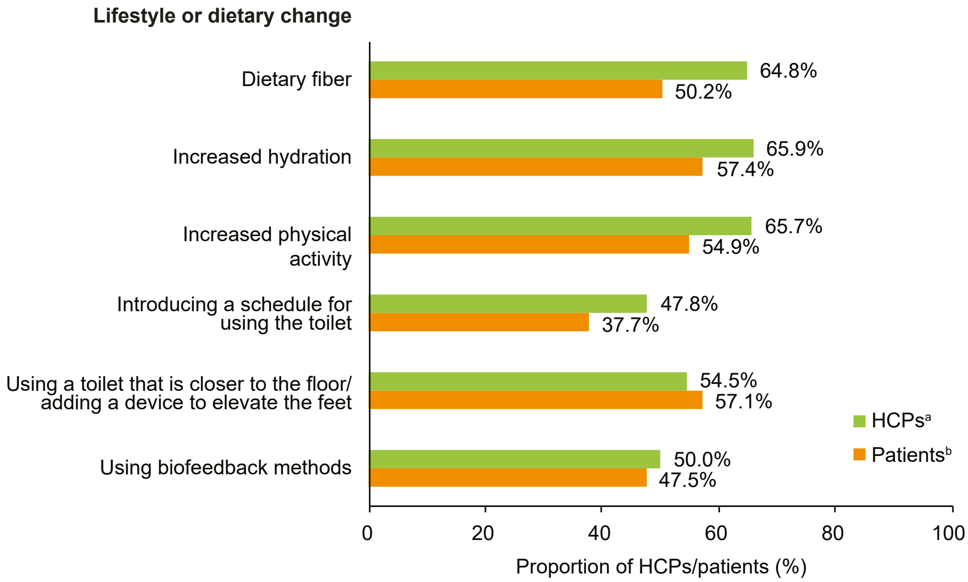

Figure 3. Proportions of patients and HCPs who considered a lifestyle or dietary modification effective in relieving symptoms of CIC. [a]N = 170. [b]Dietary fiber, N = 324; increased hydration, N = 314; increased physical activity, N = 280; introducing a schedule for using the toilet, N = 134; using a toilet that is closer to the floor/adding a device to elevate the feet, N = 99; using biofeedback methods, N = 60. Data were collected via the CRF and the patient survey. HCPs could choose a rating from a 7-point scale: 'extremely ineffective,' 'very ineffective,' 'somewhat ineffective,' 'neutral,' 'somewhat effective,' 'very effective,' and 'extremely effective;' data are presented for attributes rated as 'somewhat effective,' 'very effective,' and 'extremely effective.' Patients could choose a rating from a 7-point scale: 'not at all effective,' 'low effectivity,' 'slightly effective,' 'neutral,' 'moderately effective,' 'very effective,' and 'extremely effective;' data are presented for attributes rated as 'moderately effective,' 'very effective,' and 'extremely effective.'

HCPs perceived linaclotide more positively in terms of safety than the 3 prescription comparators. One factor that may potentially be contributing to this is the multiple dosing strengths available to HCPs when prescribing linaclotide.[10] Statistical testing was not performed for these analyses, and therefore, any comparisons made between the HCPs' perceptions of the 4 prescription medications are descriptive. Notably, lubiprostone, plecanatide, and prucalopride are not currently FDA-approved for patients aged <18 years with CIC.[11–13] Therefore, assessing these therapeutics in a pediatric population is not currently possible. Real-world data on the pediatric CIC treatment landscape, from the perspectives of patients and their HCPs, are needed.

Overall, more than 80% of patients were somewhat to completely satisfied with the prescription medications available for CIC; this is considerably higher than previously reported in the literature.[8,24] A US patient questionnaire conducted in 2016 reported less than half (41%) of patients with CIC were satisfied or completely satisfied with their branded prescription medication owing to lack of efficacy and side effects.[8] This 2016 study was conducted before the US FDA approval of plecanatide and prucalopride[12,13]; thus, the notable increase in patient satisfaction observed in our study could therefore be attributed to the availability and efficacy of additional prescription medication options for CIC.

This study has multiple strengths, including the presentation of real-world US treatment data from a large patient population with insights from a diverse HCP population, including from multiple specialties. Another strength of this study is that patients were enrolled prospectively as the next eligible patients with whom the HCP consulted, which ensured a heterogeneous patient population and generalizability of the data. Limited patient inclusion and exclusion criteria also allowed an assessment of a wide spectrum of CIC, including patients with milder symptoms.

The limitations of this study include potential selection bias which arose from some data only being available from consulting patients who chose to complete the survey. These individuals may be more engaged than the general CIC population. No statistical testing was performed, and therefore, the analyses were descriptive. In addition, patients completed a paper survey, which presents a higher likelihood of missed questions than online surveys.[25] Recall and information bias can exist with self-reported research,[26] but matching of some of the CRF data with the patient survey responses helped to mitigate these risks. The surveys and CRF were designed specifically for this study and have not been validated for future/repeated use. Some patients included in the CRF analysis did not return the completed patient survey. Finally, standardized data collection techniques and consistent, neutral questioning helped to further mitigate the risk of information bias.

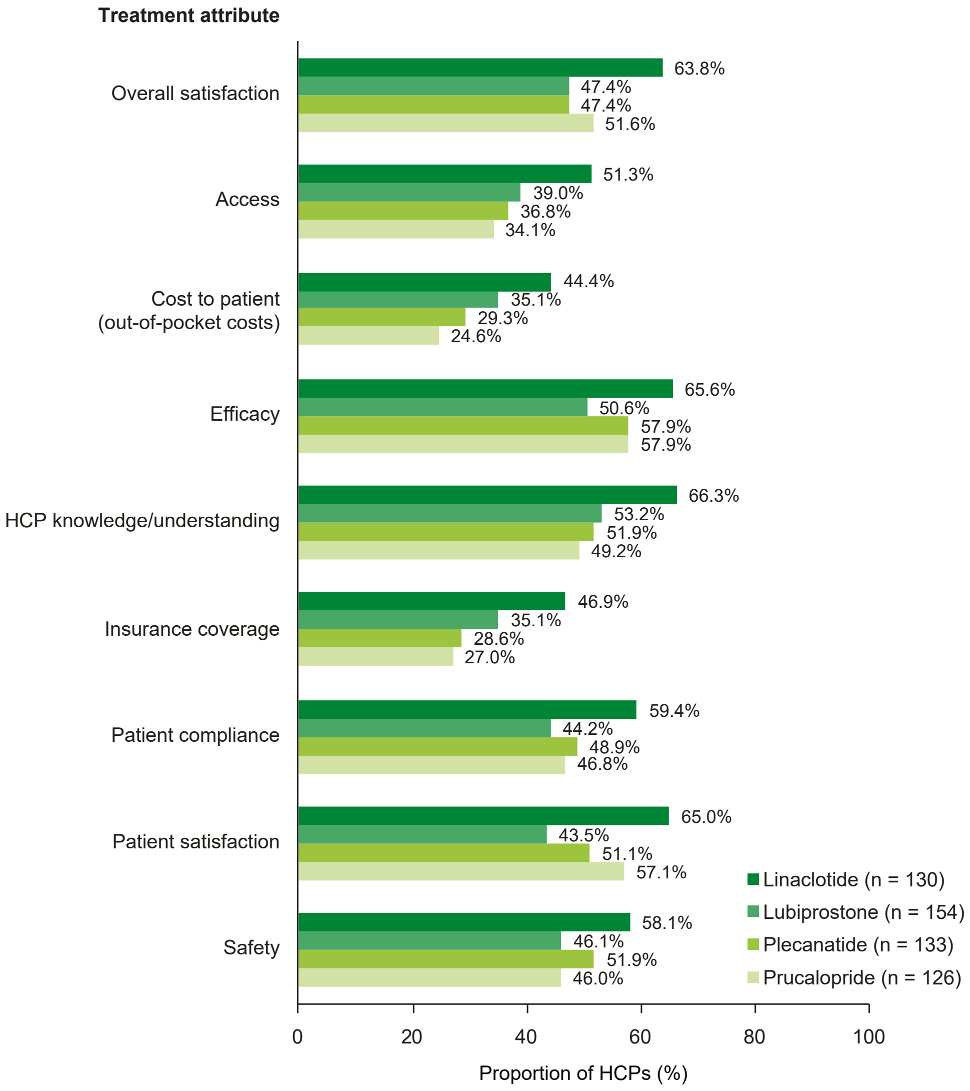

Figure 4. Proportions of HCPs who were positive about a CIC prescription medication, overall and by treatment attribute. Data were collected via the HCP survey. HCPs could choose ratings from a 7-point scale: 'very negative,' 'negative,' 'somewhat negative,' 'neutral,' 'somewhat positive,' 'positive,' and 'very positive.' Data are reported as the proportion of HCPs who rated the attribute as 'positive' or 'very positive.'

Conclusion

HCPs were generally found to follow current CIC clinical treatment guidelines by escalating CIC management from lifestyle/dietary modifications and OTC treatments to prescription medications. Most patients were satisfied with CIC treatment options, particularly with prescription medications. HCPs were generally more satisfied than patients with the use of lifestyle modifications in the treatment of CIC. Approximately 40% of the patients who had never received prescription medications for CIC were clinically eligible to do so, which may indicate wider accessibility issues. Patients also identified a lack of awareness of prescription medications and limited insurance coverage as common barriers to receiving these medications, whereas HCPs identified patient out-of-pocket costs and the complexity of getting insurance coverage as common barriers to prescribing medications. These findings highlight the importance of patient and HCP education and cost discussions at the policy level to improve access to prescription therapies for patients with CIC.

Supplementary Materials

Material associated with this article can be found in the online version at https://doi.org/10.1016/j.gastha.2026.100900.

References

1. Suares NC, Ford AC. Prevalence of, and risk factors for, chronic idiopathic constipation in the community: systematic review and meta-analysis. Am J Gastroenterol 2011;106:1582–1591.
2. Lacy BE, Mearin F, Chang L, et al. Bowel disorders. Gastroenterology 2016;150:1393–1407.
3. Rome Foundation. Rome IV diagnostic criteria for FGIDs. https://theromefoundation.org/rome-iv/rome-iv-criteria/. Accessed February 5, 2025.

4. Oh SJ, Fuller G, Patel D, et al. Chronic constipation in the United States: results from a population-based survey assessing healthcare seeking and use of pharmacotherapy. Am J Gastroenterol 2020;115:895–905.
5. Nag A, Martin SA, Mladsi D, et al. The humanistic and economic burden of chronic idiopathic constipation in the USA: a systematic literature review. Clin Exp Gastroenterol 2020;13:255–265.
6. Herrick LM, Spalding WM, Saito YA, et al. A case-control comparison of direct healthcare-provider medical costs of chronic idiopathic constipation and irritable bowel syndrome with constipation in a community-based cohort. J Med Econ 2017;20:273–279.
7. Cai Q, Buono JL, Spalding WM, et al. Healthcare costs among patients with chronic constipation: a retrospective claims analysis in a commercially insured population. J Med Econ 2014;17:148–158.
8. Harris LA, Horn J, Kissous-Hunt M, et al. The better understanding and recognition of the disconnects, experiences, and needs of patients with chronic idiopathic constipation (BURDEN-CIC) study: results of an online questionnaire. Adv Ther 2017;34:2661–2673.
9. Chang L, Chey WD, Imdad A, et al. American Gastroenterological Association-American College of Gastroenterology clinical practice guideline: pharmacological management of chronic idiopathic constipation. Gastroenterology 2023;164:1086–1106.
10. AbbVie. LINZESS (linaclotide). Highlights of prescribing information. www.accessdata.fda.gov/drugsatfda_docs/label/2023/202811s021lbl.pdf. Accessed July 31, 2025.
11. Takeda Pharmaceuticals. AMITIZA (lubiprostone). Highlights of prescribing information. www.accessdata.fda.gov/drugsatfda_docs/label/2020/021908s018lbl.pdf. Accessed July 31, 2025.
12. Synergy Pharmaceuticals. TRULANCE (plecanatide). Highlights of prescribing information. www.accessdata.fda.gov/drugsatfda_docs/label/2017/208745lbl.pdf. Accessed July 31, 2025.
13. Shire. MOTEGRITY (prucalopride). Highlights of prescribing information. www.accessdata.fda.gov/drugsatfda_docs/label/2020/210166s002lbl.pdf. Accessed July 31, 2025.
14. Lacy BE, Schey R, Shiff SJ, et al. Linaclotide in chronic idiopathic constipation patients with moderate to severe abdominal bloating: a randomized, controlled trial. PLoS One 2015;10:e0134349.
15. Johanson JF, Morton D, Geenen J, et al. Multicenter, 4-week, double-blind, randomized, placebo-controlled trial of lubiprostone, a locally-acting type-2 chloride channel activator, in patients with chronic constipation. Am J Gastroenterol 2008;103:170–177.
16. Camilleri M, Kerstens R, Rykx A, et al. A placebo-controlled trial of prucalopride for severe chronic constipation. N Engl J Med 2008;358:2344–2354.
17. DeMicco M, Barrow L, Hickey B, et al. Randomized clinical trial: efficacy and safety of plecanatide in the treatment of chronic idiopathic constipation. Therap Adv Gastroenterol 2017;10:837–851.
18. US WorldMeds. ZELNORM (tegaserod). Highlights of prescribing information. www.accessdata.fda.gov/drugsatfda_docs/label/2019/021200Orig1s015lbl.pdf. Accessed February 5, 2025.
19. Ardelyx, Inc. IBSRELA (tenapanor). Highlights of prescribing information. www.accessdata.fda.gov/drugsatfda_docs/label/2019/211801s000lbl.pdf. Accessed February 5, 2025.
20. AXCAN Pharma US, Inc. BENTYL (dicyclomine hydrochloride). Highlights of prescribing information. https://www.accessdata.fda.gov/drugsatfda_docs/label/2011/007409s041lbl.pdf. Accessed March 5, 2025.
21. Mutual Pharmaceutical Company, Inc. COLCRYS (colchicine). Highlights of prescribing information. https://www.accessdata.fda.gov/drugsatfda_docs/label/2009/022351lbl.pdf. Accessed November 11, 2025.
22. Rao SSC, Brenner DM. Evidence-based treatment recommendations for OTC management of chronic constipation. J Am Assoc Nurse Pract 2022;34:1041–1044.
23. Shah ED, Staller K, Nee J, et al. Evaluating the impact of cost on the treatment algorithm for chronic idiopathic constipation: cost-effectiveness analysis. Am J Gastroenterol 2021;116:2118–2127.
24. Taylor DCA, Abel JL, Martin C, et al. Comprehensive assessment of patients with irritable bowel syndrome with constipation and chronic idiopathic constipation using deterministically linked administrative claims and patient-reported data: the Chronic Constipation and IBS-C Treatment and Outcomes Real-World Research Platform (CONTOR). J Med Econ 2020;23:1072–1083.
25. Ebert JF, Huibers L, Christensen B, et al. Paper- or web-based questionnaire invitations as a method for data collection: cross-sectional comparative study of differences in response rate, completeness of data, and financial cost. J Med Internet Res 2018;20:24.
26. Althubaiti A. Information bias in health research: definition, pitfalls, and adjustment methods. J Multidiscip Healthc 2016;9:211–217.

Received May 2, 2025. Accepted February 11, 2026.

Correspondence:
Address correspondence to: Darren M. Brenner, MD, Feinberg School of Medicine, Northwestern University, 676 N Saint Clair Ave, Chicago, Illinois 60611. e-mail: darren.brenner@nm.org.

Authors' Contributions:
Joanna de Courcy: Concept and design; acquisition of data; statistical analysis; provision of study materials or patients; and administrative, technical, or logistical support. Jeanne Jiang: Concept and design, obtaining funding, and supervision. Mei Lu: Concept and design, obtaining funding, and supervision. Neil Reynolds: Concept and design; acquisition of data; statistical analysis; provision of study materials or patients; and administrative, technical, or logistical support. Teresa Taylor-Whiteley: Concept and design; acquisition of data; statistical analysis; provision of study materials or patients; and administrative, technical, or logistical support. Brian Terreri: Concept and design, obtaining funding, and supervision. All authors: Analysis and interpretation of data, drafting of the manuscript, and critical revision of paper for important intellectual content. The analysis, interpretation, preparation, and decision to submit the manuscript for publication was the responsibility of all authors.

Conflicts of Interest:
These authors disclose the following: Darren M. Brenner has received consultancy, speaker, or advisory board fees from AbbVie, Alnylam Pharmaceuticals, Anji Pharmaceuticals, Ardelyx, Bayer, Blueprint Medicine, CinPhloro, Entrinsic Bioscience, Gemelli Biotech, Ironwood Pharmaceuticals, Laborie, Mahana Therapeutics, Salix Pharmaceuticals, Takeda Pharmaceuticals, and Vibrant Pharma; has received nonvested stock options for Owlstone Medical and SideBy Care; and is on the board of directors for the International Foundation for Gastrointestinal Disorders. Baharak Moshiree has received research grant support from Alfasigma, Atmo Biosciences, CinDome,

CinPhloro, Restalsis Health, Inc, and Takeda Pharmaceuticals; has received advisory board fees from AbbVie, AbbVie/Ironwood Pharmaceuticals, Allergan, Ardelyx, and Takeda Pharmaceuticals; and is an inventor with patents from Atrium Health and the University of Miami on small bowel aspiration capsule and Blue Muffin meal. Joanna de Courcy, Neil Reynolds, and Teresa Taylor-Whiteley are employees of Adelphi Real World and received funding from Takeda Pharmaceuticals USA, Inc, for designing and conducting this study. Jeanne Jiang, Mei Lu, and Brian Terreri are employees of Takeda Pharmaceuticals USA, Inc, and are stockholders of Takeda Pharmaceutical Company Limited. Eric D. Shah has received consultancy fees from Ardelyx, Laborie, Mahana Therapeutics, Mylan, NeurAxis, Salix Pharmaceuticals, Sanofi, and Takeda Pharmaceuticals; has equity in Neuraxis; and holds a patent with the University of Michigan.

Funding:
Medical writing support was provided by Jessica Boles, PhD, of PharmaGenesis London, London, UK, and was funded by Takeda Pharmaceuticals USA, Inc." with "This study was funded by Takeda Pharmaceuticals USA, Inc. Medical writing support was provided by Jessica Boles, PhD, of PharmaGenesis London, London, UK." and provide grant sponsor link to "Takeda Pharmaceuticals USA, Inc.

Ethical Statement:
The protocol (22-ADRW-164) was determined by the Pearl Institutional Review Board to be exempt from ethical review on July 10, 2022, in accordance with FDA 21 CFR 56.104 and DHHS 45 CFR 46.104(b)(2): (2) Tests, Surveys, Interviews.

Data Transparency Statement:
Takeda does not plan to share the data supporting the results reported in this article.

Reporting Guidelines:
STROBE.

Gastro Hep Advances, Volume 5

Supplemental information

Treatment Patterns, Perceptions, Barriers, and Costs in Patients With Chronic Idiopathic Constipation in the United States

Darren M. Brenner, Baharak Moshiree, Joanna de Courcy, Neil Reynolds, Teresa Taylor-Whiteley, Jeanne Jiang, Mei Lu, Brian Terreri, and Eric D. Shah

Supplementary Material

Treatment Patterns, Perceptions, Barriers, and Costs in Patients with Chronic Idiopathic Constipation in the United States

Darren M. Brenner,[1,*] Baharak Moshiree,[2,*] Joanna de Courcy,[3] Neil Reynolds,[3] Teresa Taylor-Whiteley,[3] Jeanne Jiang,[4] Mei Lu,[4] Brian Terreri,[4] and Eric D. Shah[5]

[1]Feinberg School of Medicine, Northwestern University, Chicago, Illinois; [2]Atrium Health, Wake Forest University School of Medicine, Charlotte, North Carolina; [3]Adelphi Real World, Bollington, UK; [4]Takeda Pharmaceuticals USA, Inc., Lexington, Massachusetts; and [5]Division of Gastroenterology and Hepatology, University of Michigan, Ann Arbor, Michigan

*Denotes co-first authorship.

Supplementary Figure 1. Study design.

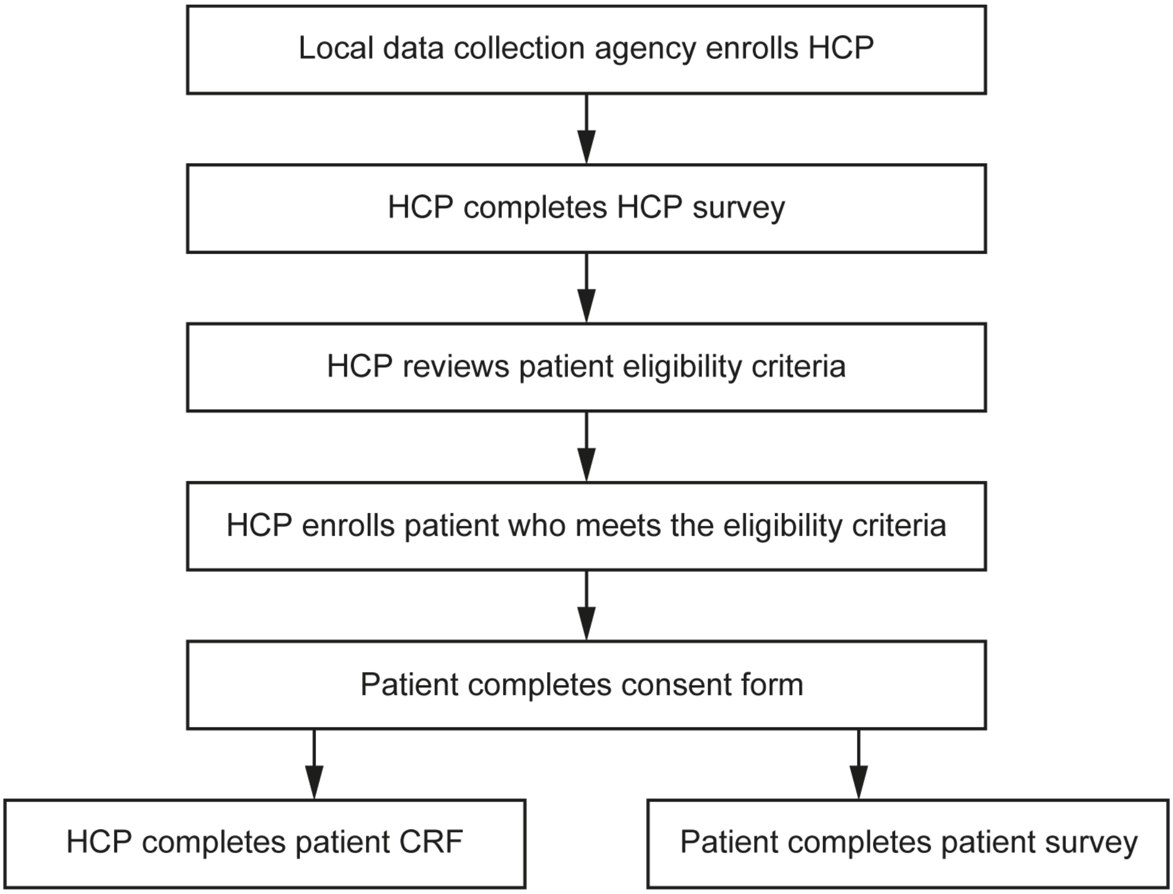

CRF, case report form; HCP, health-care professional.

Supplementary Table 1. Recommendations of CIC treatments by HCPs at first, second, third, and fourth lines in an ideal world, stratified by HCP specialty

Treatment, n (%)[a]	Total (N = 170)	General gastroenterologist (n = 53)	Motility specialist (n = 12)	Primary care physician (n = 64)	Advanced practice provider (n = 41)
**First-line treatment**					
Increased dietary fiber	151 (88.8)	43 (81.1)	12 (100.0)	58 (90.6)	38 (92.7)
Increased hydration	139 (81.8)	36 (67.9)	10 (83.3)	55 (85.9)	38 (92.7)
Increased physical activity	133 (78.2)	37 (69.8)	9 (75.0)	51 (79.7)	36 (87.8)
Introducing a schedule for using the toilet	98 (57.6)	31 (58.5)	7 (58.3)	32 (50.0)	28 (68.3)
Using a toilet that is closer to the floor/adding a device to elevate the feet	86 (50.6)	24 (45.3)	9 (75.0)	27 (42.2)	26 (63.4)
Bulk-forming laxatives	82 (48.2)	29 (54.7)	7 (58.3)	27 (42.2)	19 (46.3)
Using biofeedback methods	63 (37.1)	16 (30.2)	7 (58.3)	21 (32.8)	19 (46.3)
Osmotic laxatives	49 (28.8)	23 (43.4)	5 (41.7)	14 (21.9)	7 (17.1)
Stimulant laxatives	27 (15.9)	11 (20.8)	3 (25.0)	7 (10.9)	6 (14.6)
Lubricant laxatives	25 (14.7)	12 (22.6)	3 (25.0)	6 (9.4)	4 (9.8)
Lactulose	22 (12.9)	10 (18.9)	3 (25.0)	4 (6.3)	5 (12.2)
Hyoscyamine	14 (8.2)	8 (15.1)	1 (8.3)	5 (7.8)	0 (0.0)
Linaclotide	11 (6.5)	5 (9.4)	2 (16.7)	2 (3.1)	2 (4.9)
Plecanatide	9 (5.3)	4 (7.5)	1 (8.3)	3 (4.7)	1 (2.4)
Lubiprostone	7 (4.1)	5 (9.4)	0 (0.0)	1 (1.6)	1 (2.4)
Misoprostol	6 (3.5)	5 (9.4)	0 (0.0)	0 (0.0)	1 (2.4)
Prucalopride	6 (3.5)	4 (7.5)	1 (8.3)	1 (1.6)	0 (0.0)
Pyridostigmine	2 (1.2)	2 (3.8)	0 (0.0)	0 (0.0)	0 (0.0)
**Second-line treatment**					
Osmotic laxatives	87 (51.2)	20 (37.7)	6 (50.0)	33 (51.6)	28 (68.3)
Bulk-forming laxatives	71 (41.8)	21 (39.6)	6 (50.0)	22 (34.4)	22 (53.7)
Stimulant laxatives	68 (40.0)	22 (41.5)	5 (41.7)	21 (32.8)	20 (48.8)

Treatment, n (%)[a]	Total (N = 170)	General gastroenterologist (n = 53)	Motility specialist (n = 12)	Primary care physician (n = 64)	Advanced practice provider (n = 41)
Lubricant laxatives	63 (37.1)	16 (30.2)	4 (33.3)	22 (34.4)	21 (51.2)
Lactulose	49 (28.8)	16 (30.2)	7 (58.3)	15 (23.4)	11 (26.8)
Using biofeedback methods	48 (28.2)	12 (22.6)	6 (50.0)	15 (23.4)	15 (36.6)
Linaclotide	39 (22.9)	19 (35.8)	4 (33.3)	12 (18.8)	4 (9.8)
Lubiprostone	37 (21.8)	19 (35.8)	6 (50.0)	11 (17.2)	1 (2.4)
Using a toilet that is closer to the floor/adding a device to elevate the feet	36 (21.2)	12 (22.6)	4 (33.3)	9 (14.1)	11 (26.8)
Increased physical activity	31 (18.2)	14 (26.4)	6 (50.0)	5 (7.8)	6 (14.6)
Hyoscyamine	31 (18.2)	14 (26.4)	5 (41.7)	4 (6.3)	8 (19.5)
Plecanatide	29 (17.1)	14 (26.4)	3 (25.0)	7 (10.9)	5 (12.2)
Increased hydration	27 (15.9)	12 (22.6)	4 (33.3)	8 (12.5)	3 (7.3)
Introducing a schedule for using the toilet	27 (15.9)	11 (20.8)	5 (41.7)	6 (9.4)	5 (12.2)
Increased dietary fiber	25 (14.7)	11 (20.8)	4 (33.3)	6 (9.4)	4 (9.8)
Prucalopride	24 (14.1)	13 (24.5)	4 (33.3)	3 (4.7)	4 (9.8)
Pyridostigmine	12 (7.1)	5 (9.4)	2 (16.7)	3 (4.7)	2 (4.9)
Misoprostol	11 (6.5)	3 (5.7)	4 (33.3)	3 (4.7)	1 (2.4
**Third-line treatment**					
Plecanatide	68 (40.0)	23 (43.4)	4 (33.3)	22 (34.4)	19 (46.3)
Lubiprostone	67 (39.4)	14 (26.4)	4 (33.3)	23 (35.9)	26 (63.4)
Linaclotide	66 (38.8)	16 (30.2)	3 (25.0)	25 (39.1)	22 (53.7)
Lactulose	57 (33.5)	17 (32.1)	3 (25.0)	18 (28.1)	19 (46.3)
Prucalopride	56 (32.9)	19 (35.8)	3 (25.0)	14 (21.9)	20 (48.8)
Hyoscyamine	53 (31.2)	14 (26.4)	5 (41.7)	18 (28.1)	16 (39.0)
Stimulant laxatives	44 (25.9)	10 (18.9)	5 (41.7)	14 (21.9)	15 (36.6)
Misoprostol	43 (25.3)	13 (24.5)	4 (33.3)	12 (18.8)	14 (34.1)
Lubricant laxatives	42 (24.7)	13 (24.5)	5 (41.7)	9 (14.1)	15 (36.6)
Pyridostigmine	36 (21.2)	14 (26.4)	4 (33.3)	8 (12.5)	10 (24.4)

Treatment, n (%)[a]	Total (N = 170)	General gastroenterologist (n = 53)	Motility specialist (n = 12)	Primary care physician (n = 64)	Advanced practice provider (n = 41)
Osmotic laxatives	27 (15.9)	7 (13.2)	4 (33.3)	7 (10.9)	9 (22.0)
Using biofeedback methods	27 (15.9)	12 (22.6)	3 (25.0)	5 (7.8)	7 (17.1)
Increased hydration	25 (14.7)	11 (20.8)	6 (50.0)	6 (9.4)	2 (4.9)
Increased physical activity	19 (11.2)	10 (18.9)	3 (25.0)	4 (6.3)	2 (4.9)
Increased dietary fiber	16 (9.4)	5 (9.4)	3 (25.0)	5 (7.8)	3 (7.3)
Using a toilet that is closer to the floor/adding a device to elevate the feet	16 (9.4)	7 (13.2)	2 (16.7)	5 (7.8)	2 (4.9)
Bulk-forming laxatives	17 (10.0)	6 (11.3)	4 (33.3)	4 (6.3)	3 (7.3)
Introducing a schedule for using the toilet	14 (8.2)	4 (7.5)	3 (25.0)	5 (7.8)	2 (4.9)
**Fourth-line treatment**					
Pyridostigmine	84 (49.4)	19 (35.8)	5 (41.7)	26 (40.6)	34 (82.9)
Prucalopride	76 (44.7)	19 (35.8)	5 (41.7)	30 (46.9)	22 (53.7)
Misoprostol	74 (43.5)	19 (35.8)	5 (41.7)	21 (32.8)	29 (70.7)
Plecanatide	58 (34.1)	13 (24.5)	5 (41.7)	18 (28.1)	22 (53.7)
Lubiprostone	57 (33.5)	15 (28.3)	6 (50.0)	15 (23.4)	21 (51.2)
Linaclotide	55 (32.4)	16 (30.2)	4 (33.3)	17 (26.6)	18 (43.9)
Hyoscyamine	53 (31.2)	13 (24.5)	6 (50.0)	13 (20.3)	21 (51.2)
Lactulose	34 (20.0)	4 (7.5)	5 (41.7)	13 (20.3)	12 (29.3)
Using biofeedback methods	20 (11.8)	11 (20.8)	3 (25.0)	2 (3.1)	4 (9.8)
Lubricant laxatives	19 (11.2)	9 (17.0)	1 (8.3)	6 (9.4)	3 (7.3)
Stimulant laxatives	18 (10.6)	6 (11.3)	2 (16.7)	6 (9.4)	4 (9.8)
Introducing a schedule for using the toilet	16 (9.4)	6 (11.3)	5 (41.7)	3 (4.7)	2 (4.9)
Increased dietary fiber	15 (8.8)	7 (13.2)	3 (25.0)	4 (6.3)	1 (2.4)
Using a toilet that is closer to the floor/adding a device to elevate the feet	15 (8.8)	8 (15.1)	1 (8.3)	5 (7.8)	1 (2.4)
Increased hydration	14 (8.2)	6 (11.3)	3 (25.0)	4 (6.3)	1 (2.4)
Increased physical activity	12 (7.1)	6 (11.3)	3 (25.0)	2 (3.1)	1 (2.4)
Osmotic laxatives	11 (6.5)	5 (9.4)	3 (25.0)	2 (3.1)	1 (2.4)

Treatment, n (%)[a]	Total (N = 170)	General gastroenterologist (n = 53)	Motility specialist (n = 12)	Primary care physician (n = 64)	Advanced practice provider (n = 41)
Bulk-forming laxatives	9 (5.3)	3 (5.7)	1 (8.3)	4 (6.3)	1 (2.4)

Data were collected via the HCP survey. For each line of treatment, HCPs could select more than one treatment. Data are ordered by total frequency.

[a]Tegaserod and tenapanor are indicated for the treatment of irritable bowel syndrome with constipation in adult women aged < 65 years and adults, respectively, dicyclomine hydrochloride is indicated for the treatment of functional bowel/irritable bowel syndrome in adults, and colchicine is indicated for the treatment of gout flares and Familial Mediterranean Fever in adults and children aged ≥ 4 years; hence, these prescription medications were excluded from this table.[1-4]

CIC, chronic idiopathic constipation; HCP, health-care professional.

Supplementary Table 2. Rating of importance of attributes of CIC treatments by HCPs

Treatment attribute, n (%)	HCPs (N = 170)						
	**Not at all important**	**Low importance**	**Slightly important**	**Neutral**	**Moderately important**	**Very important**	**Extremely important**
Aligns with treatment guidelines	0 (0.0)	3 (1.8)	5 (2.9)	21 (12.4)	61 (35.9)	60 (35.3)	20 (11.8)
Ease of access	0 (0.0)	0 (0.0)	6 (3.5)	11 (6.5)	47 (27.6)	62 (36.5)	44 (25.9)
Ease of treatment administration	0 (0.0)	1 (0.6)	2 (1.2)	17 (10.0)	56 (32.9)	67 (39.4)	27 (15.9)
Effective relief of pain	0 (0.0)	0 (0.0)	5 (2.9)	13 (7.6)	37 (21.8)	75 (44.1)	40 (23.5)
Effective relief of straining	0 (0.0)	0 (0.0)	2 (1.2)	13 (7.6)	55 (32.4)	66 (38.8)	34 (20.0)
Effective relief of symptoms	0 (0.0)	0 (0.0)	1 (0.6)	13 (7.6)	36 (21.2)	74 (43.5)	46 (27.1)
Familiarity/experience	0 (0.0)	3 (1.8)	11 (6.5)	28 (16.5)	50 (29.4)	58 (34.1)	20 (11.8)
Frequency of treatment administration	0 (0.0)	6 (3.5)	13 (7.6)	18 (10.6)	66 (38.8)	45 (26.5)	22 (12.9)
Improvement in sensations of incomplete evacuation	0 (0.0)	1 (0.6)	5 (2.9)	15 (8.8)	55 (32.4)	64 (37.6)	30 (17.6)
Improvement in stool consistency	0 (0.0)	2 (1.2)	9 (5.3)	11 (6.5)	64 (37.6)	56 (32.9)	28 (16.5)
Increased quality of life	0 (0.0)	0 (0.0)	2 (1.2)	15 (8.8)	28 (16.5)	59 (34.7)	66 (38.8)
Long-term efficacy	0 (0.0)	0 (0.0)	4 (2.4)	9 (5.3)	36 (21.2)	77 (45.3)	44 (25.9)
Low level of monitoring for mental health adverse events/drug reactions	1 (0.6)	2 (1.2)	7 (4.1)	31 (18.2)	43 (25.3)	63 (37.1)	23 (13.5)
Low level of monitoring for physical adverse events/drug reactions	0 (0.0)	3 (1.8)	9 (5.3)	16 (9.4)	50 (29.4)	70 (41.2)	22 (12.9)
Low level of patient support needed	0 (0.0)	0 (0.0)	8 (4.7)	34 (20.0)	56 (32.9)	52 (30.6)	20 (11.8)
Maintains patient's ability to perform tasks/activities	0 (0.0)	0 (0.0)	8 (4.7)	17 (10.0)	43 (25.3)	65 (38.2)	37 (21.8)

Treatment attribute, n (%)	HCPs (N = 170)						
	Not at all important	Low importance	Slightly important	Neutral	Moderately important	Very important	Extremely important
Mode of treatment administration	0 (0.0)	1 (0.6)	12 (7.1)	23 (13.5)	60 (35.3)	55 (32.4)	19 (11.2)
Patient compliance	0 (0.0)	0 (0.0)	3 (1.8)	19 (11.2)	38 (22.4)	72 (42.4)	38 (22.4)
Rapid onset of action	1 (0.6)	2 (1.2)	10 (5.9)	21 (12.4)	54 (31.8)	59 (34.7)	23 (13.5)
Reasonable cost–benefit ratio	1 (0.6)	0 (0.0)	7 (4.1)	14 (8.2)	47 (27.6)	65 (38.2)	36 (21.2)
Reducing the chance of fecal impaction	0 (0.0)	1 (0.6)	7 (4.1)	13 (7.6)	49 (28.8)	62 (36.5)	38 (22.4)
Safe for short- and long-term use	0 (0.0)	1 (0.6)	5 (2.9)	13 (7.6)	34 (20.0)	63 (37.1)	54 (31.8)

Data were collected via the HCP survey.

CIC, chronic idiopathic constipation; HCP, health-care professional.

Supplementary Table 3. Rating of importance of attributes of CIC treatments by patients

**Treatment attribute, n (%)**	**Patients (N = 230)**						
	**Extremely unimportant**	**Very unimportant**	**Somewhat unimportant**	**Neutral**	**Somewhat important**	**Very important**	**Extremely important**
Affordable treatment (n = 228)	1 (0.4)	1 (0.4)	2 (0.9)	13 (5.7)	27 (11.8)	64 (28.1)	120 (52.6)
Ease of treatment administration (n = 227)	0 (0.0)	4 (1.8)	7 (3.1)	13 (5.7)	28 (12.3)	71 (31.3)	104 (45.8)
Effective relief of symptoms (n = 229)	0 (0.0)	1 (0.4)	1 (0.4)	16 (7.0)	19 (8.3)	78 (34.1)	114 (49.8)
HCP recommendation based on patient's previous experience (n = 227)	0 (0.0)	2 (0.9)	9 (4.0)	32 (14.1)	36 (15.9)	59 (26.0)	89 (39.2)
Low number of side effects (n = 229)	0 (0.0)	3 (1.3)	1 (0.4)	16 (7.0)	32 (14.0)	63 (27.5)	114 (49.8)
Safe for short- and long-term use (n = 229)	0 (0.0)	1 (0.4)	6 (2.6)	11 (4.8)	34 (14.8)	59 (25.8)	118 (51.5)

Data were collected via the patient survey.

CIC, chronic idiopathic constipation.

Supplementary Table 4. Rating of effectiveness of lifestyle or dietary modifications in relieving symptoms of CIC by HCPs and patients

Lifestyle/dietary modification, n (%)	CRF (N = 368)						
	**Extremely ineffective**	**Very ineffective**	**Somewhat ineffective**	**Neutral**	**Somewhat effective**	**Very effective**	**Extremely effective**
Dietary fiber (n = 324)	6 (1.9)	20 (6.2)	47 (14.5)	38 (11.7)	112 (34.6)	72 (22.2)	26 (8.0)
Increased hydration (n = 314)	3 (1.0)	12 (3.8)	43 (13.7)	46 (14.6)	103 (32.8)	68 (21.7)	36 (11.5)
Increased physical activity (n = 280)	2 (0.7)	14 (5.0)	33 (11.8)	33 (11.8)	103 (36.8)	55 (19.6)	26 (9.3)
Introducing schedule for using the toilet (n = 134)	3 (2.2)	10 (7.5)	19 (14.2)	33 (24.6)	43 (32.1)	16 (11.9)	5 (3.7)
Using a toilet that is closer to the floor/adding a device to elevate the feet (n = 99)	1 (1.0)	1 (1.0)	10 (10.1)	27 (27.3)	35 (35.4)	15 (15.2)	4 (4.0)
Using biofeedback methods (n = 60)	1 (1.7)	7 (11.7)	8 (13.3)	9 (15.0)	17 (28.3)	9 (15.0)	4 (6.7)
	**Patients (N = 230)**						
	**Not at all effective**	**Low effectivity**	**Slightly effective**	**Neutral**	**Moderately effective**	**Very effective**	**Extremely effective**
Dietary fiber (n = 215)	8 (3.7)	26 (12.1)	41 (19.1)	32 (14.9)	65 (30.2)	29 (13.5)	14 (6.5)
Increased hydration (n = 216)	6 (2.8)	17 (7.9)	37 (17.1)	32 (14.8)	59 (27.3)	37 (17.1)	28 (13.0)
Increased physical activity (n = 184)	7 (3.8)	19 (10.3)	28 (15.2)	29 (15.8)	53 (28.8)	29 (15.8)	19 (10.3)
Introducing schedule for using the toilet (n = 77)	7 (9.1)	9 (11.7)	12 (15.6)	20 (26.0)	25 (32.5)	2 (2.6)	2 (2.6)
Using a toilet that is closer to the floor/adding a device to elevate the feet (n = 70)	2 (2.9)	4 (5.7)	11 (15.7)	13 (18.6)	25 (35.7)	12 (17.1)	3 (4.3)
Using biofeedback methods (n = 40)	5 (12.5)	3 (7.5)	5 (12.5)	8 (20.0)	9 (22.5)	8 (20.0)	2 (5.0)

Data were collected via the CRF and the patient survey.

CIC, chronic idiopathic constipation; CRF, case report form; HCP, health-care professional**.**

Supplementary Table 5. Perceptions of four CIC prescription medications: linaclotide, lubiprostone, plecanatide, and prucalopride by HCPs

Treatment attribute, n (%)[a]	Very negative	Negative	Somewhat negative	Neutral	Somewhat positive	Positive	Very positive
**Overall satisfaction**							
Linaclotide	0 (0.0)	1 (0.6)	3 (1.9)	17 (10.6)	37 (23.1)	72 (45.0)	30 (18.8)
Lubiprostone	0 (0.0)	0 (0.0)	11 (7.1)	24 (15.6)	46 (29.9)	57 (37.0)	16 (10.4)
Plecanatide	0 (0.0)	0 (0.0)	4 (3.0)	14 (10.5)	52 (39.1)	45 (33.8)	18 (13.5)
Prucalopride	1 (0.8)	0 (0.0)	8 (6.3)	20 (15.9)	32 (25.4)	50 (39.7)	15 (11.9)
**Access**							
Linaclotide	1 (0.6)	6 (3.8)	7 (4.4)	23 (14.4)	41 (25.6)	59 (36.9)	23 (14.4)
Lubiprostone	1 (0.6)	7 (4.5)	13 (8.4)	29 (18.8)	44 (28.6)	42 (27.3)	18 (11.7)
Plecanatide	4 (3.0)	5 (3.8)	18 (13.5)	19 (14.3)	38 (28.6)	33 (24.8)	16 (12.0)
Prucalopride	3 (2.4)	6 (4.8)	21 (16.7)	16 (12.7)	37 (29.4)	32 (25.4)	11 (8.7)
**Cost to patient (out-of-pocket costs)**							
Linaclotide	1 (0.6)	7 (4.4)	15 (9.4)	26 (16.3)	40 (25.0)	55 (34.4)	16 (10.0)
Lubiprostone	2 (1.3)	8 (5.2)	19 (12.3)	32 (20.8)	39 (25.3)	36 (23.4)	18 (11.7)
Plecanatide	5 (3.8)	7 (5.3)	23 (17.3)	18 (13.5)	41 (30.8)	26 (19.5)	13 (9.8)
Prucalopride	5 (4.0)	8 (6.3)	24 (19.0)	24 (19.0)	34 (27.0)	22 (17.5)	9 (7.1)
**Efficacy**							
Linaclotide	0 (0.0)	0 (0.0)	0 (0.0)	13 (8.1)	42 (26.3)	74 (46.3)	31 (19.4)
Lubiprostone	0 (0.0)	0 (0.0)	6 (3.9)	14 (9.1)	56 (36.4)	62 (40.3)	16 (10.4)
Plecanatide	0 (0.0)	0 (0.0)	3 (2.3)	15 (11.3)	38 (28.6)	59 (44.4)	18 (13.5)
Prucalopride	1 (0.8)	0 (0.0)	6 (4.8)	14 (11.1)	32 (25.4)	57 (45.2)	16 (12.7)
**HCP knowledge/understanding**							
Linaclotide	0 (0.0)	1 (0.6)	5 (3.1)	13 (8.1)	35 (21.9)	64 (40.0)	42 (26.3)
Lubiprostone	0 (0.0)	0 (0.0)	5 (3.2)	21 (13.6)	46 (29.9)	49 (31.8)	33 (21.4)
Plecanatide	0 (0.0)	0 (0.0)	6 (4.5)	20 (15.0)	38 (28.6)	44 (33.1)	25 (18.8)
Prucalopride	1 (0.8)	0 (0.0)	5 (4.0)	21 (16.7)	37 (29.4)	39 (31.0)	23 (18.3)
**Insurance coverage**							
Linaclotide	2 (1.3)	6 (3.8)	12 (7.5)	19 (11.9)	46 (28.8)	54 (33.8)	21 (13.1)
Lubiprostone	3 (1.9)	6 (3.9)	17 (11.0)	28 (18.2)	46 (29.9)	36 (23.4)	18 (11.7)
Plecanatide	5 (3.8)	8 (6.0)	22 (16.5)	22 (16.5)	38 (28.6)	25 (18.8)	13 (9.8)

Treatment attribute, n (%)[a]	Very negative	Negative	Somewhat negative	Neutral	Somewhat positive	Positive	Very positive
Prucalopride	3 (2.4)	9 (7.1)	26 (20.6)	21 (16.7)	33 (26.2)	26 (20.6)	8 (6.3)
**Patient compliance**							
Linaclotide	0 (0.0)	1 (0.6)	7 (4.4)	12 (7.5)	45 (28.1)	64 (40.0)	31 (19.4)
Lubiprostone	0 (0.0)	4 (2.6)	12 (7.8)	20 (13.0)	50 (32.5)	55 (35.7)	13 (8.4)
Plecanatide	0 (0.0)	1 (0.8)	4 (3.0)	17 (12.8)	46 (34.6)	44 (33.1)	21 (15.8)
Prucalopride	1 (0.8)	0 (0.0)	7 (5.6)	22 (17.5)	37 (29.4)	42 (33.3)	17 (13.5)
**Patient satisfaction**							
Linaclotide	0 (0.0)	0 (0.0)	4 (2.5)	14 (8.8)	38 (23.8)	73 (45.6)	31 (19.4)
Lubiprostone	0 (0.0)	1 (0.6)	10 (6.5)	22 (14.3)	54 (35.1)	52 (33.8)	15 (9.7)
Plecanatide	0 (0.0)	0 (0.0)	2 (1.5)	13 (9.8)	50 (37.6)	50 (37.6)	18 (13.5)
Prucalopride	1 (0.8)	0 (0.0)	6 (4.8)	18 (14.3)	29 (23.0)	60 (47.6)	12 (9.5)
**Safety**							
Linaclotide	0 (0.0)	0 (0.0)	5 (3.1)	15 (9.4)	47 (29.4)	61 (38.1)	32 (20.0)
Lubiprostone	0 (0.0)	0 (0.0)	4 (2.6)	20 (13.0)	59 (38.3)	52 (33.8)	19 (12.3)
Plecanatide	0 (0.0)	0 (0.0)	3 (2.3)	17 (12.8)	44 (33.1)	49 (36.8)	20 (15.0)
Prucalopride	1 (0.8)	1 (0.8)	4 (3.2)	22 (17.5)	40 (31.7)	43 (34.1)	15 (11.9)

Data were collected via the HCP survey.

[a]Linaclotide (n = 160), lubiprostone (n = 154), plecanatide (n = 133) and prucalopride (n = 126).

CIC, chronic idiopathic constipation; HCP, health-care professional.

Supplementary Table 6. Patient satisfaction of four CIC prescription medications they were currently receiving: linaclotide, lubiprostone, plecanatide, and prucalopride

Current prescription medication, n (%)[a]	Completely dissatisfied	Very dissatisfied	Somewhat dissatisfied	Neither satisfied nor dissatisfied	Somewhat satisfied	Very satisfied	Completely satisfied
Linaclotide	0 (0.0)	1 (1.4)	3 (4.1)	4 (5.5)	16 (21.9)	39 (53.4)	10 (13.7)
Lubiprostone	0 (0.0)	0 (0.0)	0 (0.0)	0 (0.0)	6 (24.0)	15 (60.0)	4 (16.0)
Plecanatide	0 (0.0)	1 (3.4)	2 (6.9)	0 (0.0)	4 (13.8)	15 (51.7)	7 (24.1)
Prucalopride	0 (0.0)	0 (0.0)	1 (7.1)	1 (7.1)	4 (28.6)	6 (42.9)	2 (14.3)

Data were collected via the patient survey.

[a]Linaclotide (n = 73), lubiprostone (n = 25), plecanatide (n = 29) and prucalopride (n = 14).

CIC, chronic idiopathic constipation.

Supplementary Table 7. A list of the survey questions completed by the HCPs in this study

**Survey question**	**Response**
HCP demographics and patient management	
What is your primary specialty?	• General gastroenterologist<br>• Primary care physician/family practitioner<br>• Gastroenterologist motility specialist<br>• Nurse practitioner working in gastroenterology<br>• Nurse practitioner working in primary care<br>• Physician assistant working in gastroenterology<br>• Physician assistant working in primary care
In the last 12 months, approximately how many patients have you personally managed for CIC?	[Free text response]
How long have you been managing patients with CIC?	• Less than 1 year<br>• 1–3 years<br>• 3–5 years<br>• 5 years+
In what type of setting do you see patients?	• Community hospital<br>• Academic center<br>• Private center<br>• Government or VA hospital<br>• Outpatient clinic/office/family medicine center<br>• Long-term care facility or nursing home<br>• Other
In which of the following settings is your practice based?	• Urban<br>• Rural
Perceptions and expectations of medicine	
Importance in CIC management (respond for each of the following):<br>• aligns with treatment guidelines<br>• ease of access<br>• ease of treatment administration	• Not at all important<br>• Low importance<br>• Slightly important<br>• Neutral

• effective relief of pain<br>• effective relief of straining<br>• effective relief of symptoms<br>• familiarity/experience<br>• frequency of treatment administration<br>• improvement in sensations of incomplete evacuation<br>• improvement in stool consistency<br>• increased quality of life<br>• long-term efficacy<br>• low level of monitoring for mental health adverse events/ drug reactions<br>• low level of monitoring for physical adverse events/ drug reactions<br>• low level of patient support needed<br>• maintains patient's ability to perform tasks/activities<br>• mode of treatment administration<br>• patient compliance<br>• rapid onset of action<br>• reasonable cost–benefit ratio<br>• reducing the chance of fecal impaction<br>• safe for short- and long-term use	• Moderately important<br>• Very important<br>• Extremely important
Barriers to treatment	
What, if anything, prevents you prescribing treatments to CIC patients (respond for each of the following):<br>• complexity of getting insurance coverage<br>• concerns with efficacy<br>• concerns with safety<br>• delays in diagnosis of CIC<br>• lack of evidence supporting their use<br>• lack of experience prescribing certain medications<br>• negative experience using prescription medications for previous patients<br>• patient did not want to take the medication prescribed to them	[0–100][a]

• patient out-of-pocket cost for medications<br>• patient preference for over-the-counter options<br>• patient reluctant to take any medications at all for their condition<br>• unaware of available treatment options<br>• other[b]	
Current treatment landscape	
In an ideal world, what would you choose to prescribe/recommend for each of the following lines of treatment?[c,d]<br>• First line<br>• Second line<br>• Third line<br>• Fourth line	• Bulk-forming laxatives<br>• Colchicine<br>• Dicyclomine hydrochloride<br>• Hyoscyamine<br>• Increased dietary fiber<br>• Increased hydration<br>• Increased physical activity<br>• Introducing a schedule for using the toilet<br>• Lactulose<br>• Linaclotide<br>• Lubiprostone<br>• Lubricant laxatives<br>• Misoprostol<br>• Osmotic laxatives<br>• Plecanatide<br>• Prucalopride<br>• Pyridostigmine<br>• Stimulant laxatives<br>• Tegaserod<br>• Tenapanor<br>• Using a toilet that is closer to the floor/adding a device to elevate the feet<br>• Using biofeedback methods
Perceptions and use of linaclotide, lubiprostone, plecanatide and prucalopride	

Of all your patients with CIC, what percentage: have never received prucalopride, but are clinically eligible	[Free text response]
Of all your patients with CIC, what percentage: have never received lubiprostone, but are clinically eligible	[Free text response]
Of all your patients with CIC, what percentage: have never received plecanatide, but are clinically eligible	[Free text response]
Of all your patients with CIC, what percentage: have never received linaclotide, but are clinically eligible	[Free text response]
Perception compared to other prescription medications (respond for each of the four prescription medications with each attribute):<br>• lubiprostone<br>• linaclotide<br>• plecanatide<br>• prucalopride<br><br>• access<br>• cost to patient (out-of-pocket costs)<br>• insurance coverage<br>• overall efficacy<br>• overall satisfaction<br>• patient compliance<br>• patient satisfaction<br>• safety<br>• your understanding/knowledge	• Very negative<br>• Negative<br>• Somewhat negative<br>• Neutral<br>• Somewhat positive<br>• Positive<br>• Very positive

[a]0 = issue does not prevent prescribing treatments at all; 100 = issue completely prevents prescribing a treatment; [b]There was no option in the survey for HCPs to provide further information; [c]HCPs could select more than one response; [d]Tegaserod and tenapanor are indicated for the treatment of irritable bowel syndrome with constipation in adult women aged < 65 years and in adults, respectively, dicyclomine hydrochloride is indicated for the treatment of functional bowel/irritable bowel syndrome in adults, and colchicine is indicated for the treatment of gout flares and Familial Mediterranean Fever in adults and children aged ≥ 4 years; hence, these prescription medications were excluded from these analyses.[1-4]

CIC, chronic idiopathic constipation; HCP, health-care professional; VA, Veterans Affairs.

Supplementary Table 8. A list of the survey questions completed by the patients in this study

**Survey question**	**Response**
Patient demographics	
Patient age	[Free text response]
Sex at birth	• Female<br>• Male<br>• Other<br>• Prefer not to say
Patient ethnicity	• American Indian, Indigenous American or Alaska Native<br>• Black or African American<br>• East or Southeast Asian<br>• Middle Eastern or North African<br>• Native Hawaiian or Pacific Islander<br>• South Asian (Indian subcontinent)<br>• White<br>• Other
Are you of Hispanic, Latin or Spanish origin?	• Yes<br>• No<br>• Don’t know
How would you describe your work status?	• Full-time homemaker<br>• Full-time student<br>• Part-time student<br>• Retired<br>• Unable to work due to constipation<br>• Unemployed (for reasons related to condition<br>• Working full-time (in a paid job or as a volunteer)<br>• Working part-time (in a paid job or as a volunteer)<br>• Not listed
What best describes your type of health insurance coverage?[a]	• Cobra (continuation coverage)<br>• Employer provided/sponsored insurance

	• Health insurance exchange plan<br>• Medicaid (or equivalent in your state)<br>• Medicare<br>• Medicare advantage<br>• Medicare medical savings account (MSA)<br>• Medicare part D prescription drug plan<br>• Non-Medicare retired benefit<br>• Partner/family member employer<br>• Privately arranged insurance<br>• Tricare/Veterans' health care<br>• No insurance coverage
Treatment journey and perception	
Which, if any, of the below behavioral/lifestyle modifications are you currently using to treat constipation?[a]	• Drinking more water<br>• Exercising more<br>• Introducing a schedule for using the toilet<br>• Taking in more dietary fiber<br>• Using a toilet that is closer to the floor/adding a device to elevate<br>your feet<br>• Using biofeedback therapy<br>• None of the above
How effective were behavioral/lifestyle modifications in relieving the symptoms of constipation (respond for each of the following):<br>• drinking more water<br>• exercising more<br>• introducing a schedule for using the toilet<br>• taking in more dietary fiber<br>• using a toilet that is closer to the floor/adding a device to elevate your feet<br>• using biofeedback therapy	• Not all effective<br>• Low effectivity<br>• Slightly effective<br>• Neutral<br>• Moderately effective<br>• Very effective<br>• Extremely effective<br>• N/A
What prescription medications are you currently receiving for constipation?[a,b]	• Colchicine<br>• Dicyclomine hydrochloride<br>• Hyoscyamine

	• Lactulose<br>• Linaclotide<br>• Lubiprostone<br>• Misoprostol<br>• Plecanatide<br>• Prucalopride<br>• Pyridostigmine<br>• Tegaserod<br>• Tenapanor
How important is each attribute to you when thinking about the treatment of your constipation (respond for each of the following):<br>• health-care professional recommendation based on their patient's previous experience<br>• treatment doesn't have many side effects<br>• treatment is affordable<br>• treatment is easy to take<br>• treatment is safe to use (short and long term)<br>• works well in relieving symptoms (including abdominal bloating and discomfort)	• Extremely unimportant<br>• Very unimportant<br>• Somewhat unimportant<br>• Neutral<br>• Somewhat important<br>• Very important<br>• Extremely important
Which, if any, of the below have you experienced in relation to your medications/treatment to treat your constipation[a]	• Couldn't afford an appointment with a health-care provider<br>• Difficulty getting an appointment to see a health-care provider about treatment for your constipation<br>• Having to travel a long way to see a health-care provider for your constipation, meaning you see your health-care practitioner less than you should<br>• Issues accessing online/telemedicine appointments and weren't able to attend face-to-face appointments with a HCP due to COVID restrictions<br>• The medication that the health-care provider wanted to prescribe would mean you had to pay too much, so you did not take the medication<br>• The travel to the health-care provider is expensive, meaning you see your health-care practitioner less than you should

	• You did not realize that prescription treatments were available for constipation<br>• Your HCP did not know about prescription treatment options<br>• Your HCP was reluctant to prescribe a medication<br>• Your insurance did not cover your prescription medication for your constipation
The last 30 days, how much (on average) did you pay towards the expenses specifically relating to your constipation (respond for each of the following):<br>• HCP appointments<br>• co-payment for prescription medications<br>• OTC treatments<br>• transport to medical appointments<br>• tests or laboratory results<br>• emergency department visits<br>• parking fees	[Free text response]
How satisfied, if at all, are you with the current treatment you are receiving for your constipation (respond for each of the following):<br>• linaclotide<br>• lubiprostone<br>• plecanatide<br>• prucalopride	• Completely dissatisfied<br>• Very dissatisfied<br>• Somewhat dissatisfied<br>• Neither satisfied nor dissatisfied<br>• Somewhat satisfied<br>• Very satisfied<br>• Completely satisfied

[a]Patients could select more than one response; [b]Tegaserod and tenapanor are indicated for the treatment of irritable bowel syndrome with constipation in adult women aged < 65 years and in adults, respectively, dicyclomine hydrochloride is indicated for the treatment of functional bowel/irritable bowel syndrome in adults, and colchicine is indicated for the treatment of gout flares and Familial Mediterranean Fever in adults and children aged ≥ 4 years; hence, these prescription medications were excluded from these analyses.[1-4]

CIC, chronic idiopathic constipation; HCP, health-care professional; N/A, not applicable; OTC, over-the-counter.

Supplementary Table 9. First-line CIC treatments received by patients, overall and by HCP specialty

Treatment, n (%)[a]	Total (N = 303)	General gastroenterologist (n = 74)	Motility specialist (n = 30)	Primary care physician (n = 87)	Advanced practice provider (n = 112)
Bulk-forming laxatives	136 (44.9)	35 (47.3)	16 (53.3)	37 (42.5)	48 (42.9)
Osmotic laxatives	134 (44.2)	28 (37.8)	17 (56.7)	44 (50.6)	45 (40.2)
Stimulant laxatives	110 (36.3)	33 (44.6)	13 (43.3)	27 (31.0)	37 (33.0)
Linaclotide	69 (22.8)	22 (29.7)	2 (6.7)	24 (27.6)	21 (18.8)
Lubiprostone	58 (19.1)	18 (24.3)	9 (30.0)	15 (17.2)	16 (14.3)
Lubricant laxatives	44 (14.5)	11 (14.9)	9 (30.0)	12 (13.8)	12 (10.7)
Plecanatide	34 (11.2)	9 (12.2)	2 (6.7)	12 (13.8)	11 (9.8)
Prescription osmotic laxatives	25 (8.3)	3 (4.1)	3 (10.0)	13 (14.9)	6 (5.4)
Prucalopride	18 (5.9)	7 (9.5)	3 (10.0)	4 (4.6)	4 (3.6)
Hyoscyamine	13 (4.3)	1 (1.4)	0 (0.0)	7 (8.0)	5 (4.5)
Misoprostol	2 (0.7)	1 (1.4)	0 (0.0)	1 (1.1)	0 (0.0)
Pyridostigmine	2 (0.7)	2 (2.7)	0 (0.0)	0 (0.0)	0 (0.0)

Data were collected via the CRF. For each line of treatment, HCPs could select more than one treatment. Data are ordered by total frequency.
[a]Tegaserod and tenapanor are indicated for the treatment of irritable bowel syndrome with constipation in adult women aged < 65 years and in adults, respectively, dicyclomine hydrochloride is indicated for the treatment of functional bowel/irritable bowel syndrome in adults, and colchicine is indicated for the treatment of gout flares and Familial Mediterranean Fever in adults and children aged ≥ 4 years; hence, these prescription medications were excluded from this table.[1-4]
CIC, chronic idiopathic constipation; CRF, case report form; HCP, health-care professional.

Supplementary Table 10. Direct and indirect expenses associated with CIC treatment reported by patients for the past 30 days

Expense in the past 30 days (N = 230)	Cost, $, mean (SD)
HCP appointments (n = 178)	30.3 (49.4)
Co-payment for prescription medications (n = 187)	28.4 (40.0)
OTC treatments (n = 199)	24.3 (20.3)
Transport to medical appointments (n = 179)	23.5 (32.7)
Tests or laboratory results (n = 158)	22.1 (51.7)
Emergency department visits (n = 154)	7.6 (54.0)
Parking fees (n = 162)	4.1 (10.8)

Data were collected via the patient survey.

HCP, health-care professional; OTC, over-the-counter; SD, standard deviation.

References

1. US WorldMeds. ZELNORM (tegaserod). Highlights of prescribing information. www.accessdata.fda.gov/drugsatfda_docs/label/2019/021200Orig1s015lbl.pdf. Accessed March 9, 2026
2. Ardelyx, Inc. IBSRELA (tenapanor). Highlights of prescribing information. www.accessdata.fda.gov/drugsatfda_docs/label/2019/211801s000lbl.pdf. Accessed March 9, 2026
3. AXCAN Pharma US, Inc. BENTYL (dicyclomine hydrochloride). Highlights of prescribing information. https://www.accessdata.fda.gov/drugsatfda_docs/label/2011/007409s041lbl.pdf. Accessed March 9, 2026
4. Mutual Pharmaceutical Company, Inc. COLCRYS (colchicine). Highlights of prescribing information. . https://www.accessdata.fda.gov/drugsatfda_docs/label/2009/022351lbl.pdf. Accessed March 9, 2026
